# The RNA helicase DDX5 promotes viral infection via regulating N^6^-methyladenosine levels on the DHX58 and NFκB transcripts to dampen antiviral innate immunity

**DOI:** 10.1371/journal.ppat.1009530

**Published:** 2021-04-28

**Authors:** Jian Xu, Yunhong Cai, ZhenBang Ma, Bo Jiang, Wenxiao Liu, Jing Cheng, Nannan Guo, Zishu Wang, Joshua E. Sealy, Cuiping Song, Xiaojia Wang, Yongqing Li

**Affiliations:** 1 Institute of Animal Husbandry and Veterinary Medicine, Beijing Academy of Agricultural and Forestry Sciences, Beijing, P. R. China; 2 College of Animal Science and Technology, Jiangxi Agricultural University, Nanchang, Jiangxi, P. R. China; 3 The Pirbright Institute, Ash Rd, Pirbright, Woking, United Kingdom; 4 China Animal Health and Epidemiology Center, Qingdao, Shandong, P. R. China; 5 College of Veterinary Medicine, China Agricultural University, Beijing, P. R. China; Duke University Medical Center, UNITED STATES

## Abstract

Multi-functional DEAD-box helicase 5 (DDX5), which is important in transcriptional regulation, is hijacked by diverse viruses to facilitate viral replication. However, its regulatory effect in antiviral innate immunity remains unclear. We found that DDX5 interacts with the N^6^-methyladenosine (m6A) writer METTL3 to regulate methylation of mRNA through affecting the m6A writer METTL3–METTL14 heterodimer complex. Meanwhile, DDX5 promoted the m6A modification and nuclear export of transcripts DHX58, p65, and IKKγ by binding conserved UGCUGCAG element in innate response after viral infection. Stable IKKγ and p65 transcripts underwent YTHDF2-dependent mRNA decay, whereas DHX58 translation was promoted, resulting in inhibited antiviral innate response by DDX5 via blocking the p65 pathway and activating the DHX58-TBK1 pathway after infection with RNA virus. Furthermore, we found that DDX5 suppresses antiviral innate immunity in vivo. Our findings reveal that DDX5 serves as a negative regulator of innate immunity by promoting RNA methylation of antiviral transcripts and consequently facilitating viral propagation.

## Author summary

DEAD-box helicase 5 (DDX5) greatly contributes to cancer development and facilitation of viral propagation. However, how DDX5 manipulates host cell processes to facilitate replication remains poorly understood. In this study, we found DDX5 is a negative antiviral regulator through manipulating N^6^-methyladenosine (m6A) of transcripts in innate immunity. Firstly, DDX5 recruited the RNA m6A “writer” METTL3 to control the m6A writer complex, then specifically promoted m6A modification and nuclear export of DDX5 binding transcripts by binding conserved UGCUGCAG element in innate immune response, ultimately, leading to RNA decay of antiviral transcripts in a YTHDF2-dependent manner. Consequently, DDX5 played vital roles in cellular RNA metabolisms to negatively regulate innate immune response to viral infection. It is the first time to unravel DDX5 as an important component that mediates modification of N^6^-methyladenosine of mRNA in regulating innate immunity.

## Introduction

Innate immunity is one of the first barriers against pathogen invasion, and it is initiated to eliminate pathogens through the use of pattern recognition receptors (PRRs) [1]. PRRs, including several regulators in the cytoplasm and nucleus, control innate immunity by activating or degrading key components of antiviral signaling [1,2]. Until now, many cytosolic PRRs have been demonstrated to be essential for innate recognition of viral components and are involved in controlling viral infection. Several key cytosolic PRRs include Toll-like receptors, retinoic acid-inducible gene I (RIG-I)-like receptors, and nucleotide-binding domain and leucine-rich repeat-containing receptors [1,3]. Upon infection, viral nucleic acids are recognized by PRRs, which subsequently recruit the adaptor protein of antiviral signaling protein to activate TANK-binding kinase 1 (TBK1)/inhibitor- nuclear factor kappa B kinase ε (IKKε) [4]. Activated TBK1 and IKKε phosphorylate interferon regulatory factor 3/7 (IRF3/7) or p65, which translocate into the nucleus to induce type I interferon (IFN) or inflammatory cytokine production [5].

The DEAD/DEAH box helicases are a family of proteins that unwind nucleic acids and are involved in various aspects of RNA metabolism, including nuclear transcription, pre-mRNA splicing, ribosome biogenesis, nucleocytoplasmic transport, translation, RNA decay, and organellar gene expression [6,7]. Many DDX helicase members play a role in innate immunity and viral infection [8,9]. DDX58 (RIG-I) recognizes viral RNA, DDX41 recognizes intracellular DNA and bacterial cyclic dinucleotides, DDX46 entraps m6A-demethylated antiviral transcripts in the nucleus, DDX19 promotes TBK1 and IKKε degradation, DDX39A alters binding and export of antiviral transcripts, and DDX3X activates the STING-IRF7-IFN-β signaling axis [10–16]. Other DDX helicases, such as DHX9, DHX36, DDX60, and DDX24, have also been identified as receptors for viral nucleic acids to regulate innate immunity [17–20]. Nearly all DDX proteins are involved in RNA metabolism in host cells [6]; thus, it is likely that DDX helicases control innate immune responses through RNA modification. However, the mechanisms through which DDX helicases regulate RNA modification are largely unknown.

The N^6^-methyladenosine (m6A) in mRNA accounts for the most abundant mRNA internal modification and as such is an important regulatory mechanism that controls gene expression in a diversity of physiological processes [21]. The m6A methylation of mRNA occurs primarily through the m6A writer complex, which comprises the core N^6^-adenosine methyltransferase METTL3 and its adaptor METTL14 [22]; then, m6A is removed by the m6A “eraser” ALKBH5 in the nucleus, and the mRNAs are exported to the cytoplasm where binding by cytosolic reader proteins occurs to stabilize, translate, and localize mRNAs [23]. Crucially, studies have found that PRRs and cytokine receptors transduce divergent and highly interconnected signals that activate infection-specific transcription patterns to immune response and inflammation [24,25]. In recent years, the RNA m6A modification has been found to play a role in the replication of numerous viruses and immune response to viral infection [26,27]. This modification can impact viral replication and can be hijacked to evade host cell immunity [25]. However, the mechanism of m6A modification in innate immunity remains unclear. It is possible that the m6A eraser ALKBH5 could be manipulated by RNA viruses through the removal of m6A from the mRNA of antiviral signaling molecules to sequester them in the nucleus [14]. Furthermore, RNA virus replication can be regulated by the RNA m6A writer [28,29]; therefore, it is possible that the RNA m6A writer may participate in antiviral innate immunity, although its roles in this process are unknown.

DDX5 is a multi-functional RNA DEAD-box helicase, which greatly contributes to cancer development and plays roles in tumorigenesis, proliferation, differentiation, metastasis, and malignancies [30,31]. It is also associated with the replication of several viruses [32]. However, the potential mechanisms of DDX5 have remained elusive. In this study, we investigated DDX5, an important component of the pathway that mediates m6A methylation of mRNA. We show that DDX5 acts as a negative regulator of the antiviral innate response by recruiting the m6A “writer” METTL3 to promote RNA m6Amodification and nuclear export of antiviral mRNAs, which undergo YTHDF2-dependent RNA decay. Our results shed light on the mechanism of m6A RNA modification in regulating innate immune response to viral infection, which could provide insights into the development of antiviral therapeutics.

## Results

### DDX5 negatively regulates IFN-β and IL-6 production

**Fig 1:**
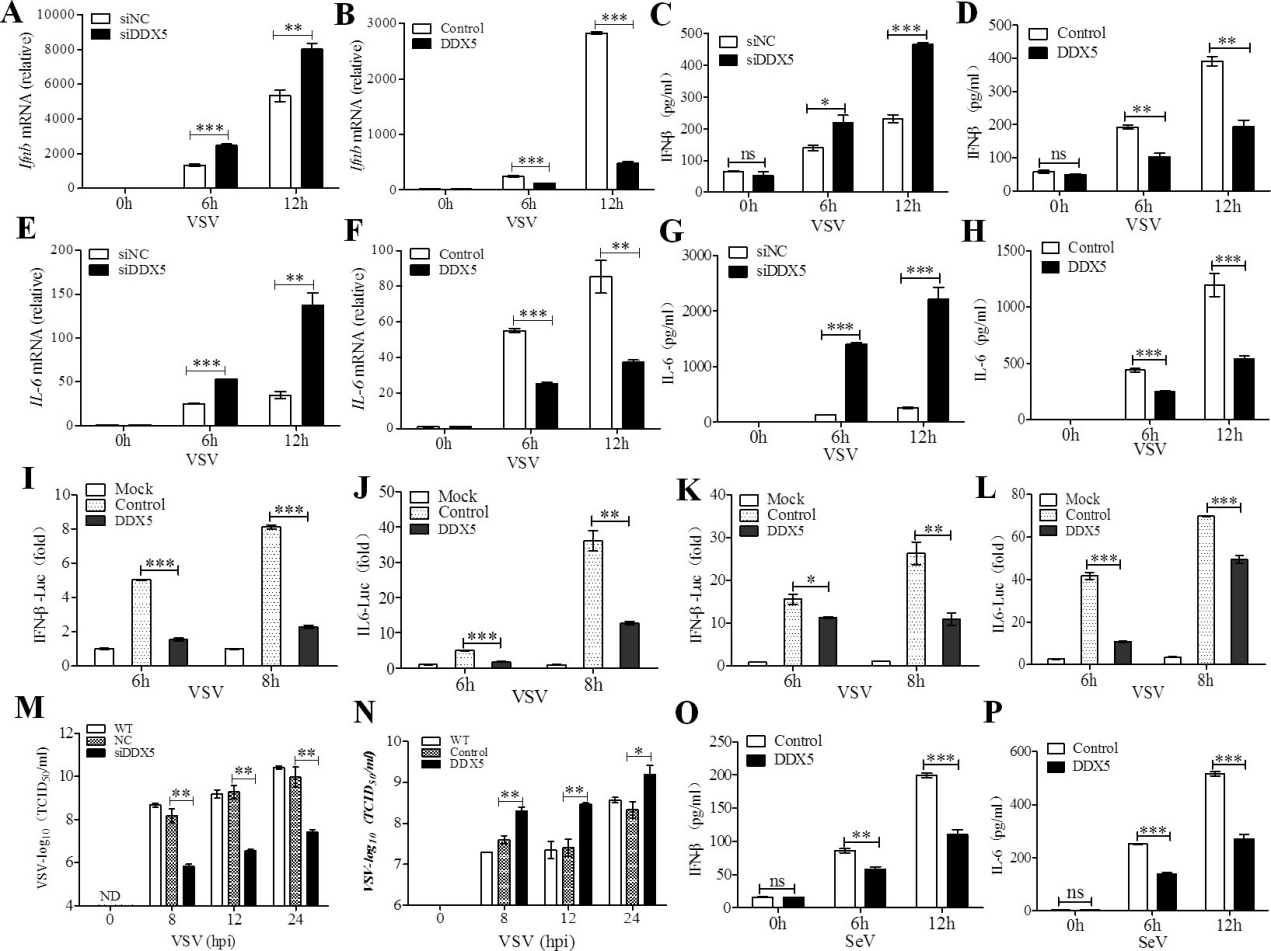
DDX5 promotes VSV replication by suppressing VSV-triggered production of IFN-β and IL-6 in MEFs. **(A, B):** qRT-PCR of Ifnb mRNA in DDX5-knockdown or -overexpressed MEFs infected with VSV for 0, 6, and 12 h. **(C, D):** ELISA of IFN-β in the supernatants of DDX5-knockdown or -overexpressed MEFs infected with VSV for 0, 6and 12 h. **(E, F):** qRT-PCR of IL-6 mRNA in DDX5-knockdown or–overexpressed MEFs infected with VSV for 0, 6, and 12 h. **(G, H):** ELISA of IL-6 in supernatants of DDX5-knockdown or -overexpressed MEFs infected with VSV for 0,6, and 12 h. **(I, J):** HEK293T cells were transfected with 500ng IFN-β (I) or IL-6 reporter (J), 20 ng Renilla-TK reporter, and 500 ng plasmid expressing DDX5 or control vector. After 24 h, cells were infected with VSV(MOI = 10). **(K, L):** MEFs were treated as described in (I, J), the luciferase activity of IFN-β (K) and IL-6 (L) were detected by the DLR Assay System. **(M, N):**TCID_50_ of VSV in DDX5-knockdown (M) or -overexpressed (N) MEFs infected with VSV for 0, 8, 12, and 24 h. **(O, P):** ELISA of IFN-β and IL-6 in supernatants of DDX5-overexpressed MEFs infected with SeV for 0,6, and 12 h. All data are mean ± SEM of biologically independent samples. Data are representative of three independent experiments. ND, not detected. ns, no significant difference. *p<0.05, **p<0.01, and ***p<0.001 (Student’s *t*-test).

Several DDX proteins have been reported to regulate innate immunity during infection [13–16]. DDX5, as a major DDX protein, has been demonstrated to regulate the replication of various viruses [32]. Thus, we aimed to determine the impact of DDX5 on the infection of mouse embryo fibroblasts (MEFs) with vesicular stomatitis virus (VSV). We found that the mRNA expression of IFN-β and IL-6, which are vital antiviral modulators of innate immune response [1,3], significantly increased in DDX5-knockdown MEFs at 6 and 12 h post-infection (hpi) with VSV (Figs S1A, 1A, 1E, and S1 Table). Conversely, IFN-β and IL-6 mRNA expression significantly decreased with DDX5 overexpression (Figs S1B, 1B, and 1F). IFN-β and IL-6 production in the culture supernatant of DDX5-knockdown MEFs also displayed a significant increase at 6 and 12 hpi (Fig 1C and 1G). In contrast, this production was significantly reduced in that of MEFs overexpressing DDX5 (Fig 1D and 1H). If DDX5 impacts the transcriptional activation of IFN-β and IL6, we investigated the promoter activity of IFN-β and IL-6 in DDX5-overexpressing HEK293T cells and MEFs, luciferase reporter assays showed that DDX5 inhibited IFN-β (Fig 1I and 1K) and IL-6 (Fig 1J and 1L) reporter activation, indicating that DDX5 negatively regulates IFN-β and IL-6 production. Furthermore, at several tested time points up to 24 hpi with VSV, replication of VSV was significantly suppressed in DDX5-knockdown MEFs, whereas it was significantly increased in DDX5-overexpressing MEFs (Fig 1M and 1N). Furthermore, IFN-β and IL-6 production also significant decreased in the DDX5-overexpressed MEF culture supernatant at 6 and 12 hpi with senda virus (SeV) (Fig 1O and 1P). Taken together, these results suggest that DDX5 was able to promote VSV replication and inhibit the production of IFN-β and IL-6.

### DDX5 interacted with METTL3

**Fig 2:**
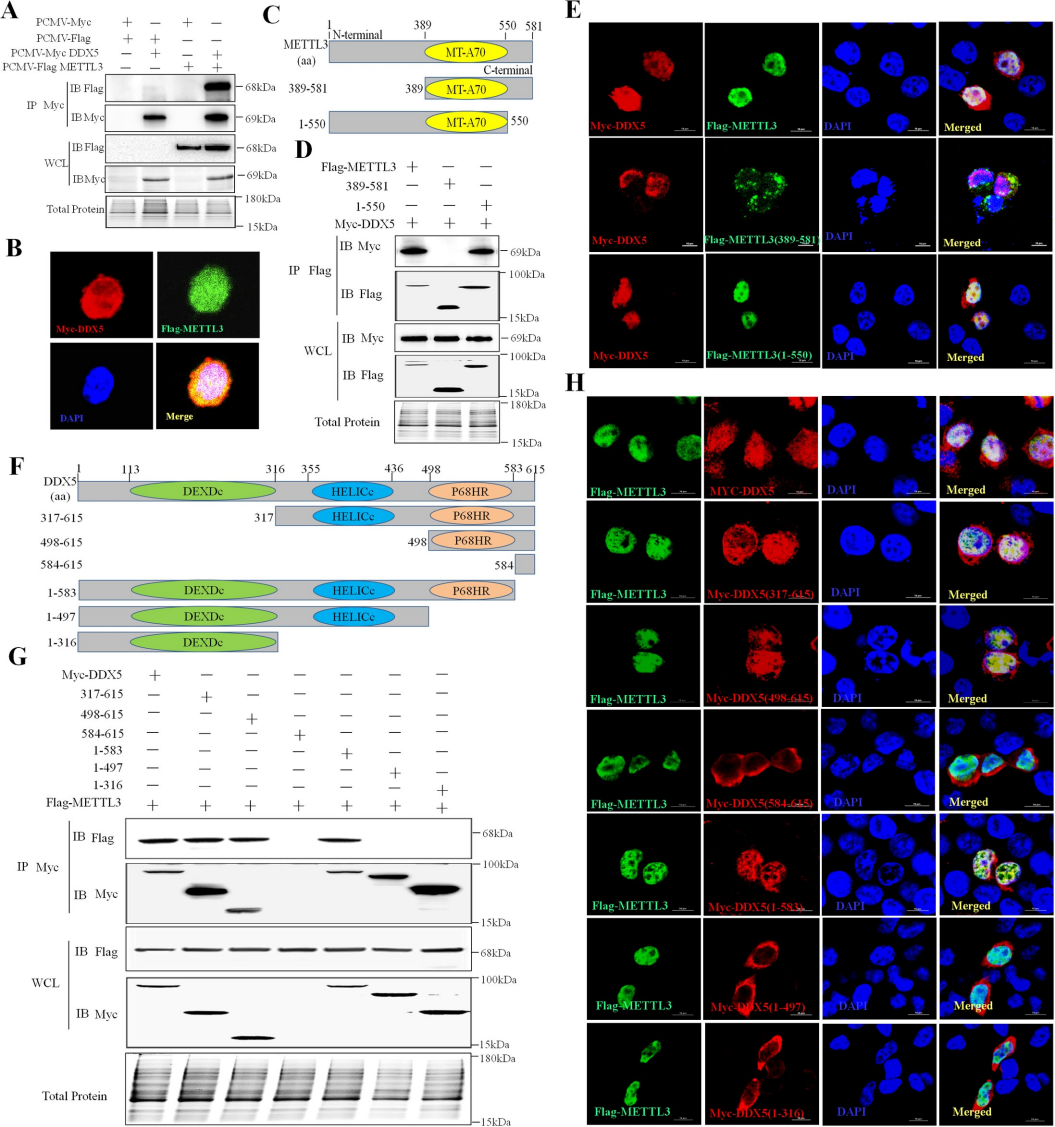
DDX5 directly interacts with METTL3. **A:** Co-IP of DDX5 and METTL3 in HEK293T cells. **B:** Co-localization of DDX5 and METTL3 in HEK293T cells cultured in slices and co-transfected with Myc-DDX5 and Flag-METTL3 plasmids. After 24 h, slices were fixed, permeabilized, and incubated with rabbit anti-Myc and mouse anti-Flag antibodies followed by Alexa Fluor 546 labeled goat anti-rabbit IgG (H+L), Alexa Fluor 488 labeled goat anti-mouse IgG (H+L). Nuclei were stained with DAPI; cells were observed by LSCM. Scale bars, 5 μm. **C:** Schematic of full-length METTL3 and its truncated mutants. **D:** Co-IP of DDX5 and METTL3 mutants in HEK293T cells. Cells were co-transfected with Myc-DDX5 and Flag-METTL3 mutants. After 24 h, cells were lysed and subjected toIP with Flag antibody, and whole-cell lysates and IP were analyzed by western blotting. **E:** Co-localization of DDX5 and METTL3 mutants in HEK293T cells. Scale bars, 10 μm. **F:** Schematic of full-length DDX5 and its truncated mutants. **G:** Co-IP of METTL3 and DDX5 mutants in HEK293T cells co-transfected with Flag-METTL3 and Myc-DDX5 mutants. After 24 h, cells were lysed and underwent IP with Myc antibody. Whole-cell lysates and IP were analyzed by western blotting. **H:** Co-localization of METTL3 and DDX5 mutants in HEK293T cells cultured and co-transfected with Flag-METTL3 and Myc-DDX5 mutant plasmids. After 24 h, slices underwent IFA. Scale bars, 10 μm.

To explore the protein-protein interactions of DDX5 in VSV-infected cells, we used pull-down assays with DDX5 from lysates of uninfected or VSV-infected MEFs or macrophages. The different protein bands were acquired and subjected to mass spectrometry to identify DDX5-associated proteins (S2 Fig). The m6A writer METTL3 was targeted in these assays on account of its significance in regulating RNA metabolism [33]. The immunoprecipitation (IP) data showed that DDX5 interacts with METTL3 and co-localizes with it in the cytoplasm and nucleus (Fig 2A and 2B). To further investigate the METTL3 protein domain involved in the interaction with DDX5, we constructed mutants of METTL3 (N-terminal deleted [1-389AA] and C-terminal deleted [551-581AA]) (Fig 2C and S2 Table). We found that METTL3 with an N-terminal deletion did not interact with DDX5, while METTL3 with a deleted MT-A70 domain or C-terminal domain had no impact on its interaction with DDX5 (Fig 2D). This indicated that DDX5 interacted with the N-terminal domain of METTL3. Furthermore, we observed co-localization of DDX5 and METTL3 mutants (Fig 2E). Nearly all C-terminal-deleted METTL3 and wild-type METTL3 co-localized with DDX5 in the nucleus, whereas N-terminal-deleted METTL3 did not co-localize with DDX5. Instead, it mostly dispersed in the cytoplasm, which verified that DDX5 interacts with the N-terminal domain of METTL3.

To determine which DDX5 domain interacts with METTL3, we constructed DDX5 mutants with deletions of DEXDc, HELICc, and P68HR (Fig 2F and S2 Table) and carried out further IP assays (Fig 2G). Compared with other mutants, P68HR domain-deleted DDX5 lost the capacity to interact with METTL3. Unlike DEXDc or HELICc domain-deleted mutants, DDX5 lacking P68HR was distributed in the cytoplasm with nearly no co-localization in the nucleus (Fig 2H), indicating that DDX5 interacted with METTL3 via its P68HR domain. Taken together, the above results demonstrated that the P68HR domain of DDX5 and the N-terminal of METTL3 are necessary for the interaction between DDX5 and METTL3.

### DDX5 regulates m6A writer complex formation and activity by recruiting METTL3 after viral infection

**Fig 3:**
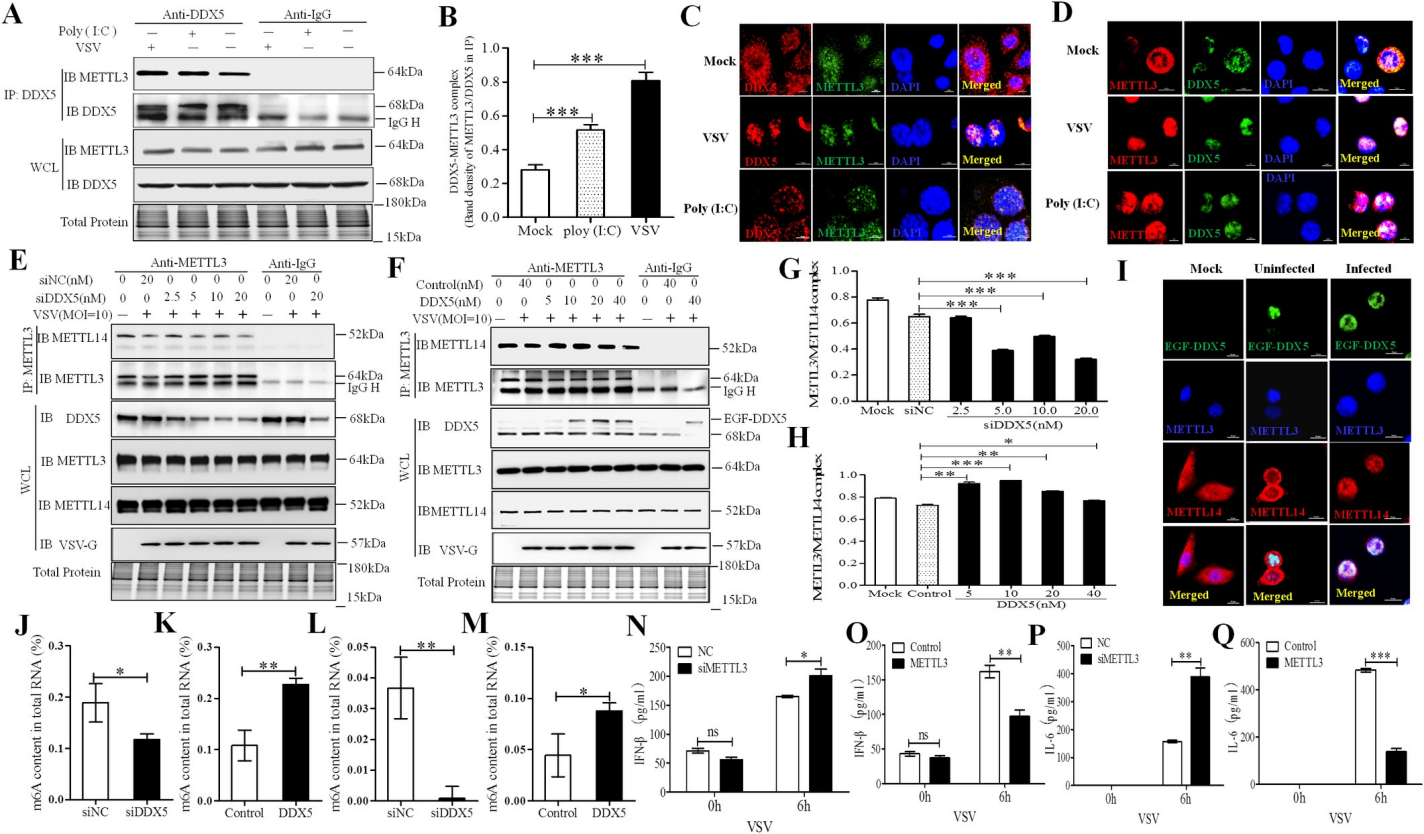
DDX5 regulated the m6A writer complex by recruiting METTL3 after VSV infection. **(A, B):** VSV promoted the interaction of DDX5 and METTL3 in MEFs. MEFs were infected with VSV or treated with Poly (I:C) for 8h and lysed with NP40 lysis buffer. Lysates were subjected to IP with DDX5 antibody or IgG. Whole-cell and IP lysates were analyzed by western blotting. The DDX5-METTL3 interaction complex was quantified by the western blot band density of METTL3/DDX5 of IP lysates. **(C, D):** Co-localization of DDX5 and METTL3 in MEFs (C) or macrophages (D) after VSV infection. MEFs/macrophages were cultured in slices and transfected with VSV or treated with Poly (I:C) for 8h. Slices were fixed, permeabilized, and incubated with rabbit anti-DDX5 and mouse anti-METTL3 antibodies followed by Alexa Fluor 546 labeled goat anti-rabbit IgG and Alexa Fluor 488 labeled goat anti-mouse IgG. Nuclei were stained with DAPI. Cells were observed by LSCM. Scale bars, 10 μm. **(E, F):** The interaction of METTL3 and METTL14 in DDX5-knockdown MEFs (E) or enhanced green fluorescent (EGF) tag fused DDX5-overexpressed MEFs (F). MEFs were transfected with different doses of DDX5 siRNA (siNC) for 48 h (E) or with different doses of EGF-DDX5 expression plasmids (control vector) for 24 h. Cells were infected with VSV (MOI = 10) for 8 h and lysed with NP40 lysis buffer, lysates were subjected to IP with rabbit METTL3 antibody or IgG. Whole-cell and IP lysates were analyzed by western blotting. **(G, H):** METTL3-METTL14 complex was quantified based on band intensity on western blot, METTL14 was normalized to METTL3 in IP assay, METTL3-METTL14 complex was determined in DDX5 knockdown IP assay (**E**) or DDX5 over-expressed IP assay (**F**). **I:** Co-localization of exogenous DDX5, METTL3, and METTL14 in VSV-infected MEFs. MEFs were cultured in slices and transfected with EGF-DDX5 expression plasmids. After 24 h, cells were infected with VSV for 8h; then, slices were fixed, permeabilized, and incubated with rabbit anti-METTL3 and mouse anti-METTL14 antibodies followed by goat anti-mouse IgG (H+L) highly cross-adsorbed secondary antibody, Alexa Fluor 647, goat anti-rabbit IgG (H+L) secondary antibody, and DyLight 405. Nuclei were stained with DAPI. Normal MEFs were used as controls. Slices were observed by LSCM. Scale bars, 10 μm. **(J, K):** The m6A content in total RNA was detected in DDX5-silenced MEFs (**J**) or DDX5-expressing MEFs (**K**) after VSV infection by ELISA. MEFs were transfected with DDX5 siRNA for 48h (**J**) or DDX5 expression plasmid for 24 h (K). Cells were infected with VSV for 8 hours used to detect m6A content in total RNA using anm6A RNA Methylation Assay Kit. **(L, M):** The m6A content in total RNA was detected in DDX5-silenced macrophages (**L**) or DDX5-expressing macrophages (**M**) after VSV infection by ELISA. Macrophages were infected with ADV1-siDDX5 (**L**) or lentiviral DDX5 for 48 h (**M**). Cells were infected with VSV for 8h and used to detect the m6A content in total RNA using anm6A RNA Methylation Assay Kit. **(N, O):** ELISA analysis of IFN-β in the supernatants of METTL3-knockdown or–overexpressed MEFs infected with VSV for 0 or 6 h. (**P, Q):** ELISA analysis of IL-6 in supernatants of METTL3-knockdown or -overexpressed MEFs infected with VSV for 0 or 6 h. All data are mean ± SEM of biologically independent samples. Data are representative of three independent experiments. ns, no significant difference. *p<0.05, **p<0.01, ***p<0.001 (Student’s *t*-test).

METTL3 is a catalytic protein that combines with the adaptor protein METTL14 to form the METTL3–METTL14 heterodimer, which constitutes the m6A writer complex to “write” m6A into mRNAs [34]. To explore the regulatory role of DDX5 on m6A in VSV-infected cells, we evaluated the interaction between DDX5 and METTL3 (Fig 3A and 3B). Compared with the results of the control group (Mock), VSV significantly enhanced the interaction between DDX5 and METTL3; meanwhile, the co-localization of DDX5 and METTL3 was more aggregated in the nuclei of VSV-infected MEFs than in those of mock control cells (Fig 3C), for further confirming this result in innate immune cells, we also observed the co-location and aggregation of DDX5 and METTL3 in macrophages which is different from MEFs in morphology (Fig 3D), all this results indicating that the interaction between DDX5 and METTL3 could be enhanced during VSV infection. This also suggests that DDX5 may adjust the RNA methylation modification activity of the m6A writer complex. We found that the METTL3-METTL14 heterodimer was significantly reduced when DDX5 was silenced using siRNA (Fig 3E and 3G), whereas overexpression of DDX5 led to an increase in heterodimers (Fig 3F and 3H). This indicated that DDX5 could regulate the RNA methylation writer complex by affecting the interaction between METTL3 and METTL14. To further confirm this result, we observed the localization of exogenous enhanced green fluorescent (EGF)-DDX5, METTL3, and METTL14 in VSV infected MEF by confocal laser-scanning microscopy (CLSM). We found that a small amount of METTL3 was co-localized with METTL14 in the nucleus of Mock control cells or VSV-uninfected cells expressing EGF-DDX5, but nearly all METTL3 co-localized with METTL14 in the nuclei of VSV-infected cells expressing EGF-DDX5 (Fig 3I). This showed that DDX5 promotes the formation of the m6A writer complex by interacting with METTL3 during VSV infection. However, whether DDX5 affected the RNA m6A level in cells after VSV infection is unknown. We found that compared with control groups, the proportion of methylated mRNA in total RNA was significantly reduced in VSV-infected DDX5-knockdown MEFs and innate immune macrophages (Fig 3J and 3L). Additionally, methylated mRNA was significantly increased in VSV-infected DDX5-overexpressing MEFs and macrophages (Figs 3K and 3M). Taken together, these data indicated that DDX5 affected RNA methylation by regulating the m6A writer complex in VSV-infected cells. To further investigate the influence of the RNA methylation writer in innate immunity, we detected the production of IFN-β and IL-6 in METTL3-knockdown or METTL3-overexpressing MEFs. We found that IFN-β and IL-6 production was significantly increased in MEFs with METTL3-knockdown (Fig 3N and 3P) and significantly decreased in those with METTL3-overexpression (Fig 3O and 3Q). These results demonstrated that DDX5 regulated RNA methylation by recruiting METTL3 to inhibit innate immunity after VSV infection.

### Identification of DDX5-bound RNAs

**Fig 4:**
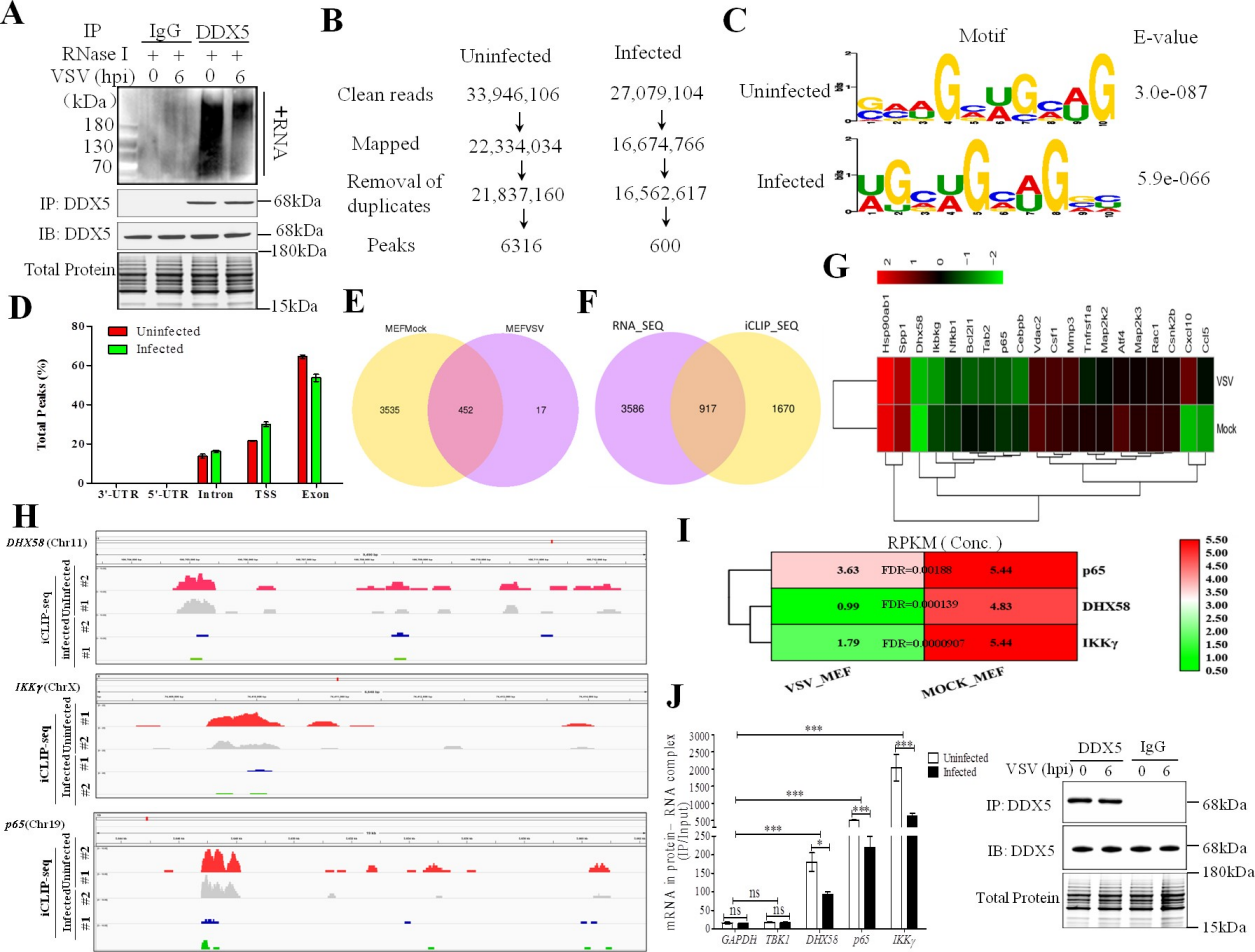
Analysis of DDX5-bound antiviral transcripts from VSV-infected MEFs by iCLIP-seq and RNA-seq technology. **A:** Immunoblot of DDX46-bound biotin-labeled RNA by iCLIP based on IgG (control) or anti-DDX5 from MEFs infected for 0 or 6 h with VSV. RNA was treated with RNase I and transferred to a PVDF membrane. Biotin-labeled RNA was detected and visualized by the chemiluminescent nucleic acid detection module. The expression levels of indicated proteins were analyzed by western blotting. **B:** iCLIP-seq alignment and processing pipeline resulting in peaks. **C:** Consensus motif of DDX5-bound RNAs (identified by E values) of the multiple-sequence alignment with the greatest enrichment under significant iCLIP-seq peaks in transcripts from uninfected or VSV-infected MEFs. **D:** Frequency of total peaks under substantial iCLIP-seq date. UTR, untranslated region; TSS, transcriptional start site. **E:** Venn diagram of different peaks in transcripts from uninfected or VSV-infected MEFs. Significantly different peaks (*p*<0.05) were analyzed using iCLIP-seq. **F:** Venn diagram of transcripts with significantly different binding to DDX5 (*p*<0.05) and significantly different expression (*p*<0.05) in uninfected or VSV-infected MEFs that were analyzed via combined iCLIP-seq and RNA-seq. **G:** Heatmap of differential expression of innate immune-associated DDX5-bound transcripts after VSV infection. Differential expression of innate immune-associated DDX5-bound transcripts was generated from iCLIP-seq and RNA-seq data (*p*<0.01). **H:** Sequencing read clusters (tracks from the IGV visualization tool for interactive exploration of genomic data sets) from iCLIP analysis of DHX58, IKKγ, and p65 in uninfected or VSV-infected (MOI = 10) MEFs, above tracks, pre-mRNA genomic loci. **I:** Heatmap of DDX5-bound transcripts in uninfected or VSV-infected MEFs. DDX5-bound transcripts were acquired by iCLIP-Seq assay. Differences in DDX5-bound transcripts were assessed based on the RPKM value. FDR, false discovery rate. **J:** Abundance of GAPDH, TBK1, DHX58, IKKγ, and p65 transcripts in DDX5–RNA complexes was determined via co-IP of DDX5 and RNA (RNA-binding protein immunoprecipitation) from MEFs infected with VSV (MOI = 10) for 0 or 6 h. The expression levels of the indicated proteins were analyzed using western blotting. The results are presented relative to the control IP of protein–RNA with input (IP/input). All data are presented as mean ± SEM of biologically independent samples. Data are representative of three independent experiments. ns, no significant difference. *p<0.05, ***p<0.001 (Student’s *t*-test).

Previous studies showed that DDX5 is an RNA-binding protein that plays an essential role in gene expression through interactions with RNA transcripts [35]. Identifying DDX5-bound RNAs would help to elucidate the relationship between innate immunity and RNA methylation; therefore, we performed individual-nucleotide resolution crosslinking IP (iCLIP) coupled with high-throughput sequencing to identify RNAs bound to DDX5. The immunoblot analysis of protein–RNA complexes immunoprecipitated with the antibody to DDX5 (anti-DDX5), but not with the control antibody to immunoglobulin G (anti-IgG), suggested that DDX5-bound RNAs were enriched after IP (Fig 4A). Furthermore, DDX5-bound RNAs were reduced in VSV-infected MEFs compared with those in uninfected MEFs. Next, cDNA libraries were generated from DDX5-bound RNAs, and deep sequencing was carried out. From two pooled iCLIP analyses of DDX5, we acquired 33 × 10^6^ and 27 × 10^6^ reads from uninfected and infected MEFs, respectively (Fig 4B); after mapping the mouse genome and removing PCR duplicates, we obtained 6316 and 600 peaks, respectively. We found that mRNAs specifically bound to DDX5 through the conserved “GAAGCUGCAG” element in uninfected MEFs, whereas in infected MEFs, mRNAs specifically bound to DDX5 at the conserved “UGCUGCAGGC” motif. All annotated genes showed that DDX5 bound to GCUGCAG in mRNA transcripts (Fig 4C). DDX5 binding involved introns (13.82% in uninfected MEFs, 16.275% in infected MEFs), the transcriptional start site (21.68% in uninfected MEFs, 30.56% in infected MEFs), and exons (64.50% in uninfected MEFs, 53.165% in infected MEFs) (Figs S3 and 4D). To understand the characteristics of peaks bound to DDX5, a Venn diagram of uninfected and infected MEFs was generated based on the iCLIP-seq analysis (Fig 4E). Comparison of 3,987 peaks from uninfected cells and 469 peaks from VSV-infected cells revealed 452 overlapping peaks. To analyze transcript-derived peaks, we also prepared Venn diagrams of transcripts that were not only significantly bound to DDX5 (*p*<0.05), but also had significantly different expression (*p*<0.05) between uninfected and infected MEFs based on iCLIP-seq coupled with RNA-seq analysis (Fig 4F). We found 4503 and 2587 transcripts in uninfected and infected cells, respectively, of which 917 transcripts overlapped between the groups according to the Venn diagram, these transcripts were not only significant different bound to DDX5 (*p*<0.01), but also significant different expression (*p*<0.01) between infected vs. non-infected cells. Using these transcripts, we then identified the major innate immunity genes from the overlapped transcripts (*p*<0.01) (Fig 4G). From the several antiviral transcripts that bound to DDX5 in infected MEFs, but not uninfected MEFs, we selected three transcripts (DHX58, p65, and IKKγ) to analyze the binding between DDX5 and transcripts in innate immunity (Fig 4H), they were likely the key transcripts were involved in VSV infection of MEFs, and previous studies have showed that DHX58 and p65 were vital proteins in innate immune response to VSV infection [3,5]. We found that the amount of DHX58, p65, and IKKγ transcripts bound to DDX5 was altered when MEFs were infected with VSV (Fig 4I). Furthermore, compared with the amount of GAPDH and TBK1 transcripts, which were not targeted to DDX5 in our study and which have been used as controls in previous studies [14,15], the amount of DHX58, p65, and IKKγ transcripts in the DDX5-RNA complex was significantly reduced in VSV-infected MEFs (Fig 4J). Taken together, these results indicate that binding of DDX5 with DHX58, p65, and IKKγ is important for regulating innate immunity.

### DDX5 impels nuclear export of antiviral transcripts by increasing m6A RNA methylation

**Fig 5:**
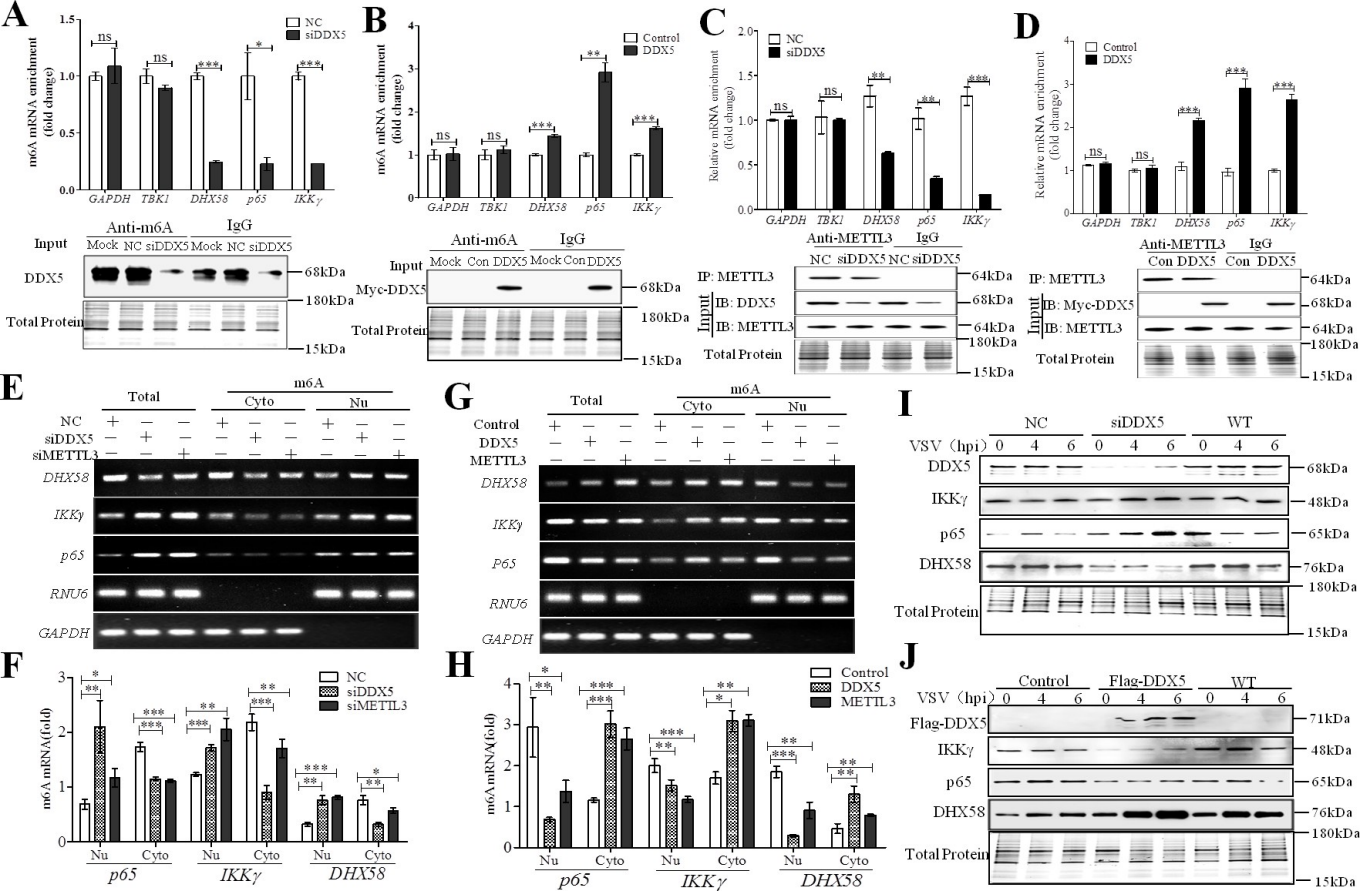
DDX5 regulated transcript expression by promoting nuclear export of m6A-methylated RNAs. **(A, B):** m6A methylation of transcripts was detected by m6A qRT-PCR in DDX5-knockdown or DDX5 overexpressed MEFs after VSV infection. MEFs were transfected with DDX5 siRNA (siNC) for 48hand infected with VSV for 6h (A), and MEFs were transfected with Myc-DDX5 expressed vector (DDX5) or Myc tag control vector (Con) for 24h and infected with VSV for 6h (B). After extracting total RNA, purifying mRNA, and removing ribosomal RNA, purified mRNA was fragmented and incubated with anti-rabbit m6A or anti-rabbit IgG-conjugated dynabeads for 4h. RNA was isolated from the solution with phenol-chloroform, and cDNA was subjected to qRT-PCR using GAPDH, TBK1, DHX58, IKKγ, and p65 primers. Results are presented relative to those obtained with NC or control groups, and the expression of all the indicated proteins was analyzed using western blotting. **(C, D):** The interaction between METTL3 and transcripts was detected through METTL3 RIP qRT-PCR in knockdown-DDX5 (C) or DDX5-expressing (D) MEFs after VSV infection. MEFs were transfected with DDX5 siRNA (siNC) for 48 h and infected with VSV for 6 h (C), and transfected with DDX5 expression plasmid (DDX5) or control vector (Con) for 24 h, infected with VSV for 6h, and subjected to METTL3 RIP qRT-PCR to detect GAPDH, TBK1, DHX58, IKKγ, and p65. Results are presented relative to those obtained with NC or control groups, and the expression of all the indicated proteins was analyzed using western blotting. **(E, F):** Nuclear transcript retention increased in DDX5-knockdown MEFs. MEFs were transfected with DDX5 siRNA (siNC), infected with VSV for 8h, and lysed to extract nuclear to cytoplasmic RNA fractions. Then, RNA was used to analyze m6A modified DHX58, IKKγ, and p65 mRNA by m6A qRT-PCR (E) with RNU6 and GAPDH as the nuclear and cytoplasmic controls, respectively. The quantitative distribution of m6A modified DHX58, IKKγ, and p65 mRNAs in DDX5-knockdown MEFs were detected by m6A qRT-PCR (F). **(G, H):** Nuclear transcript export was increased in DDX5-expressing MEFs. MEFs were transfected with DDX5 expression plasmid (control vector), infected with VSV for 8h, and lysed to extract nuclear or cytoplasmic RNA; then, RNA was used to analyze m6A modified DHX58, IKKγ, and p65 mRNA by m6A qRT-PCR (G), and the quantitative distribution of these mRNAs was detected by m6AqRT-PCR (H). **(I, J)**: Immunoblot analysis of DHX58, IKKγ, and p65 in DDX5-knockdownMEFs (I) or DDX5-expressing MEFs (J) after infection with VSV at 0, 4, and 6 h. All data are mean ± SEM of biologically independent samples. Data are representative of three independent experiments. ns, no significant difference. *p<0.05, **p<0.01, and ***p<0.001 (Student’s *t*-test).

It has been shown that previously methylated mRNA enhances mRNA export from the nucleus, a process facilitated by binding by reader proteins in the nucleus [36]. We found that DDX5 not only regulated m6A RNA methylation, but also bound directly to antiviral transcripts. We hypothesized that DDX5 regulated innate immunity by affecting the RNA methylation of antiviral transcripts in the nucleus. Results showed that, in VSV-infected cells, comparing with the transcripts GAPDH and TBK1 which were not targeted to DDX5, m6A RNA methylation of transcripts (DHX58, p65, and IKKγ) was significantly decreased when DDX5 was knocked down (Fig 5A), whereas it was significant increased when DDX5 was overexpressed (Fig 5B). This indicated that DDX5 could regulate the m6A RNA methylation of antiviral transcripts in VSV-infected cells. However, to confirm the association between DDX5 and METTL3-mediated m6A modification of the transcripts, we analyzed the abundance of DHX58, IKKγ, and p65 transcripts in METTL3/DDX5/RNA complex via METTL3 RIP-qPCR. We found that the abundance of DHX58, IKKγ, and p65 transcripts in METTL3 m6A writer was significantly decreased in DDX5 knockdown MEFs (Fig 5C) and were significantly increased in DDX5 over-expressing MEFs (Fig 5D), while there was no significant change in the abundance of GAPDH and TBK1 transcripts, which are not targeted by DDX5. We also observed the co-localization of transcripts (DHX58, IKKγ, and p65), DDX5, and METTL3 through fluorescence *in situ* hybridization (FISH) assay combined with CLSM technology (S4 Fig), which indicated that METTL3-mediated m6A modification of DHX58, IKKγ, and p65 transcripts was associated with DDX5. Studies have shown that once mRNA is modified by m6A methylation, it would be recognized by m6A reader proteins and exported to the cytoplasm from the nucleus for translation or RNA decay [22]. Therefore, we compared the cytoplasmic and nuclear abundance of m6A modified transcripts (DHX58, p65, and IKKγ). Compared with DDX5- and METTL3-non-silenced MEFs, the nuclear abundance of m6A modified DHX58, p65, and IKKγ transcripts was greater, while cytoplasmic abundance of these transcripts was lower in DDX5- and METTL3-silenced MEFs infected with VSV (Fig 5E and 5F). Meanwhile, the nuclear abundance of m6A modified DHX58, p65, and IKKγ transcripts was lower, and the cytoplasmic abundance of these transcripts was greater than that in DDX5- and METTL3-overexpressing MEFs infected with VSV (Fig 5G and 5H), suggesting that DDX5 decreased the nuclear retention and promoted nuclear export of m6A modified DHX58, p65, and IKKγ transcripts. We next found that knockdown of DDX5 increased the expression of p65 and IKKγ protein but decreased that of DHX58; meanwhile, overexpression of DDX5 decreased p65 and IKKγ protein expression but increased that of DHX58 (Fig 5I and 5J). This indicated that m6A reader proteins in the cytoplasm may play a vital role in the expression of antiviral transcripts.

### DDX5 promotes RNA decay of antiviral transcripts in a YTHDF2-dependent manner

**Fig 6:**
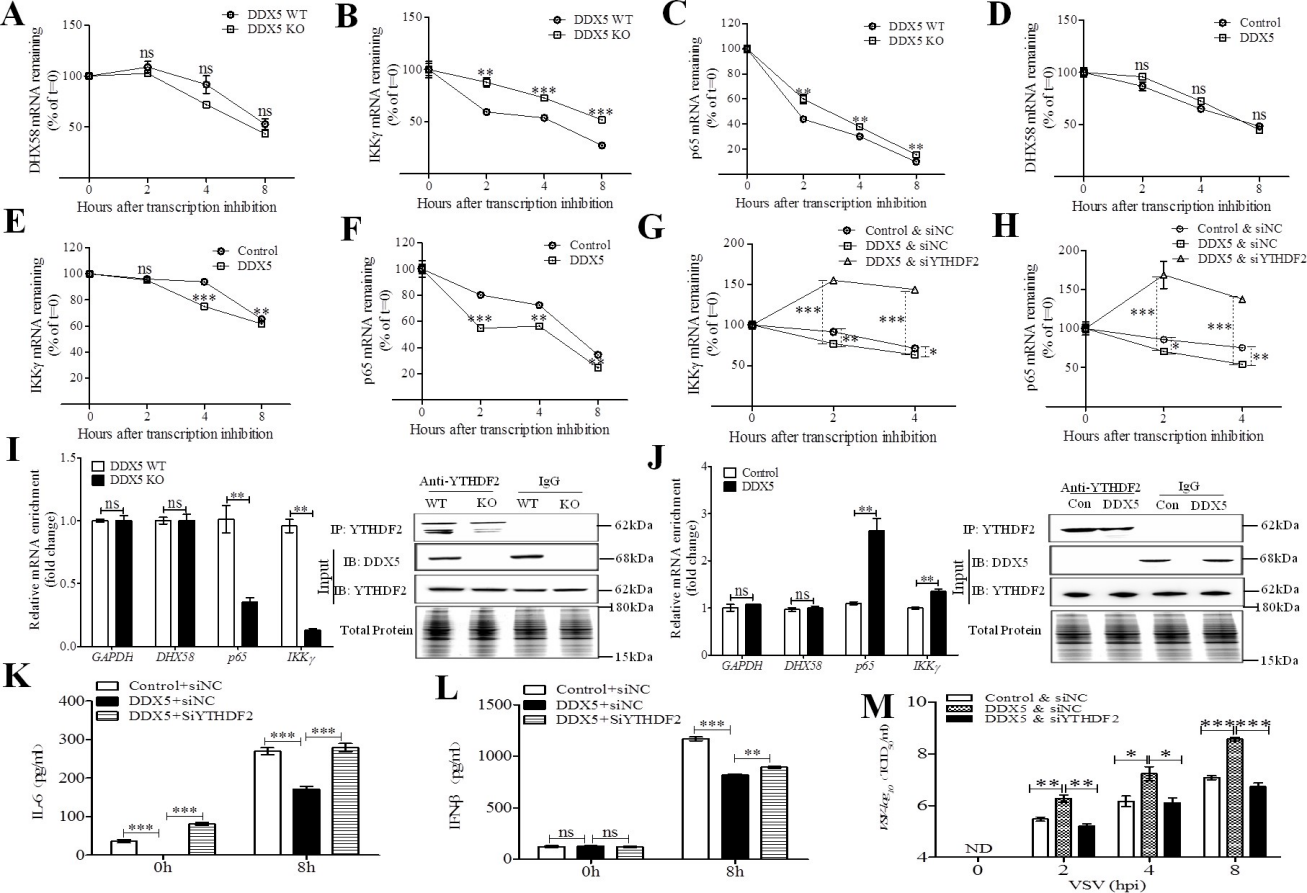
DDX5 inhibited innate immunity through promoting RNA decay of antiviral transcripts in a YTHDF2-dependent manner. **(A-C):** DHX58, IKKγ, and p65 mRNA degradation in VSV infected DDX5-knockout (KO) MEFs treated with actinomycin D at indicated times (n = 3), the DDX5 wild type (WT) MEFs as the control. Residual RNAs were normalized to 0 h. (**D-F):** DHX58, IKKγ, and p65 mRNA degradation in VSV infected DDX5-expressing MEFs treated with actinomycin D at indicated times (n = 3), the DDX5 wild type (WT) MEFs as the control. Residual RNAs were normalized to 0 h. (**G, H):** IKKγ and p65 mRNA degradation in indicated YTHDF2-silenced MEFs treated with actinomycin-D (n = 3). Residual RNAs were normalized to 0 h. MEFs were transfected with YTHDF2 siRNA (siNC) for 24h, transfected with DDX5 (control vector) for 24h, infected with VSV, and then treated with actinomycin D at indicated times (n = 3). Residual RNAs were normalized to 0 h. **(I, J):** The binding of YTHDF2 and transcripts was detected via YTHDF2 RIP qRT-PCR in DDX5 KO (I) or DDX5-expressing (J) MEFs after VSV infection. DDX5 KO MEFs were infected with VSV for 6 h (I), or MEFs were transfected with DDX5 expression plasmid (DDX5) or control vector (Con) for 24 h, then infected with VSV for 6 h (J), next all the treated MEFs were subjected to YTHDF2 RIP qRT-PCR to detect GAPDH, DHX58, IKKγ, and p65. Results are presented relative to those obtained with NC or control groups, and the expression of all the indicated proteins was analyzed by western blotting. **(K, L):** ELISA of IL-6 or IFN-β in supernatants of YTHDF2-knockdown coupled with DDX5-expressing MEFs infected with VSV for 0 or 8 h. (**M):** TCID_50_ of VSV in YTHDF2-knockdown coupled with DDX5-expressing MEFs infected with VSV for 0, 2, 4, and 8 h. All data are mean ± SEM of biologically independent samples. *n* = number of biological replicates. Data are representative of three independent experiments. ND, not detected. ns, no significant difference. *p<0.05, **p<0.01, and ***p<0.001 (Student’s *t*-test).

Previous studies have shown that m6A-methylated mRNA in the cytoplasm binds to cytosolic reader proteins that affect the stability, translation, and/or localization of mRNAs [37,38]. Therefore, we aimed to determine the stability of the abovementioned transcripts using an RNA decay assay. Compared with the control transcripts GAPDH and TBK1 (S5 Fig), the stability of p65 and IKKγ transcripts, but not that of DHX58 transcripts (Fig 6A), was significantly increased in DDX5-deficient MEFs infected with VSV after RNA synthesis was inhibited using actinomycin D (Fig 6B and 6C), whereas it was significantly decreased in DDX5-overexpressing MEFs (Fig 6E and 6F). This suggested that DDX5 exert crucial responsibility to RNA degradation of antiviral transcripts during viral infection. YTHDF2, a key reader protein for m6A methylation of RNA transcripts, promotes cytoplasmic mRNA degradation [37,39]. By knocking down YTHDF2, we could impose a delay in p65 and IKKγ mRNA degradation in DDX5-overexpressing MEFs (Figs S6, 6G, and 6H). To further confirm our hypothesis, we analyzed the binding of YTHDF2 and transcripts via RIP qRT-PCR (Fig 6I) and found that there was a significant decrease in the levels of p65 and IKKγ transcripts bound to YTHDF2 in VSV-infected DDX5 knockout (KO) MEFs, but there was no change in the levels of DHX58 transcripts and that of the negative control GAPDH bound to YTHDF2. Additionally, the binding of p65 and IKKγ transcripts to YTHDF2 was significantly increased in VSV-infected DDX5 overexpressing MEFs, while no significant difference was observed in the binding of DHX58 transcripts and GAPDH to YTHDF2 (Fig 6J), indicating that DDX5 regulated the stability of p65 and IKKγ transcripts via m6A read protein YTHDF2-mediated mRNA degradation. Silencing of YTHDF2 also blocked the inhibition of IFN-β and IL-6 production in DDX5-overexpressing MEFs (Fig 6K and 6L). This confirmed that YTHDF2-dependent RNA decay plays a vital role in innate immunity. Finally, we assayed viral replication in YTHDF2-silenced MEFs and found that replication of VSV could be largely enhanced in DDX5-overexpressing MEFs. Viral replication was reduced in DDX5-overexpressed, YTHDF2-silenced MEFs (Fig 6M). In summary, these results indicated that DDX5 promotes RNA decay of antiviral transcripts in a YTHDF2-dependent manner.

### DDX5 thwarts DHX58-TBK1 and p65 pathways in innate immunity

**Fig 7:**
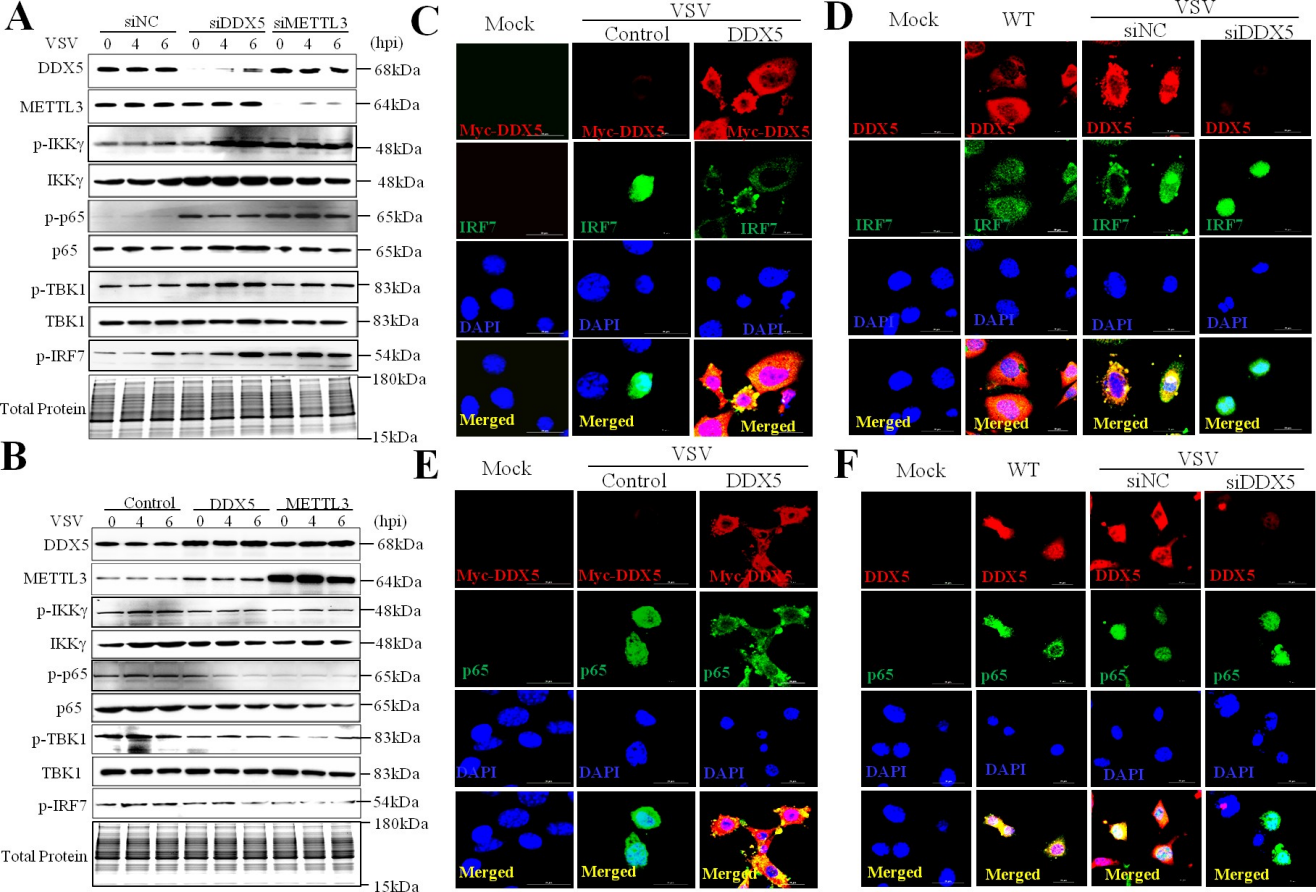
DDX5 negatively regulated DHX58-mediated TBK1 and p65 pathways in innate immunity. **(A, B):** Immunoblot of DHX58-mediated TBK1 and p65 pathways in DDX5-knockdown MEFs (A) or DDX5-expressing MEFs (B). MEFs were transfected with DDX5 siRNA or METTL3 siRNA (siNC) for 48 h (A), or with DDX5 or METTL3 expression (control vector) for 24 h (B); then, cells were infected with VSV for 0, 4, and 6h, lysed, and collected to detect DDX5, METTL3, p-IKKγ, IKKγ,p-p65,p65,p-TBK1, TBK1 and p-IRF7 via western blot. **(C, D):** Nuclear transfer of IRF7 was observed by IFA and CLSM. MEFs were transfected with DDX5-expression plasmids (control vector) for 24 h (C) or with DDX5 siRNA (siNC) for 48 h (D), infected with VSV for 8 h, and then subjected to IFA with anti-IRF7 monoclonal antibody followed by Alexa Fluor 488 F(ab′)2 fragment of goat anti-mouse IgG (H+L). Nuclei were stained with DAPI. Normal MEFs (WT) were used as the control. Slices were observed by LSCM. Scale bars, 20 μm. **(E, F):** Nuclear transfer of p65 observed by IFA and CLSM. MEFs were transfected with DDX5-expression plasmids (E) or with DDX5 siRNA (F), infected with VSV for 8h, and subjected to IFA with anti-p65 monoclonal antibody followed by Alexa Fluor 488 F (ab′)2 fragment of goat anti-mouse IgG (H+L). Nuclei were stained with DAPI. Normal MEFs (WT) were used as the control. Slices were observed by LSCM. Scale bars, 20 μm.

The production of type I interferon and inflammatory cytokines is triggered when viral nucleic acids are recognized by PRRs [1]. DHX58 has been reported as a non-signaling member of the RIG-I-like receptors, which could mediate the TBK1 and p65 pathways to activate innate immune responses against RNA viruses [40]. To further investigate the innate immunity pathways regulated by DDX5, we detected the expression of p-TBK1, p-IKKγ, p-p65, and p-IRF7 in DDX5- or METTL3-knockdown or -overexpressing MEFs after infection with VSV. Compared with control MEFs, DDX5- or METTL3-knockdown MEFs showed enhanced expression of the abovementioned proteins after VSV infection (Fig 7A), whereas those overexpressing DDX5 or METTL3 showed decreases in the expression of these phosphorylated proteins (Fig 7B). These results showed that DDX5 could block the DHX58-TBK1 and p65 pathways in innate immunity. To further confirm this conclusion, we also observed the nuclear translocation of p65 and IRF7, which have been considered as vital factors for the activation of the p65 and TBK1 pathways. Compared with the control MEFs, we found both p65 and IRF7 were dispersed and localized in the cytoplasm of DDX5-overexpressing MEFs (Fig 7C and 7E) and were aggregated and localized in the nucleus of DDX5-knockdown MEFs (Fig 7D and 7F). Taken together, DDX5 thwarts DHX58-TBK1 and p65 pathways in innate immunity.

### DDX5 negatively regulates innate immunity in vivo

**Fig 8:**
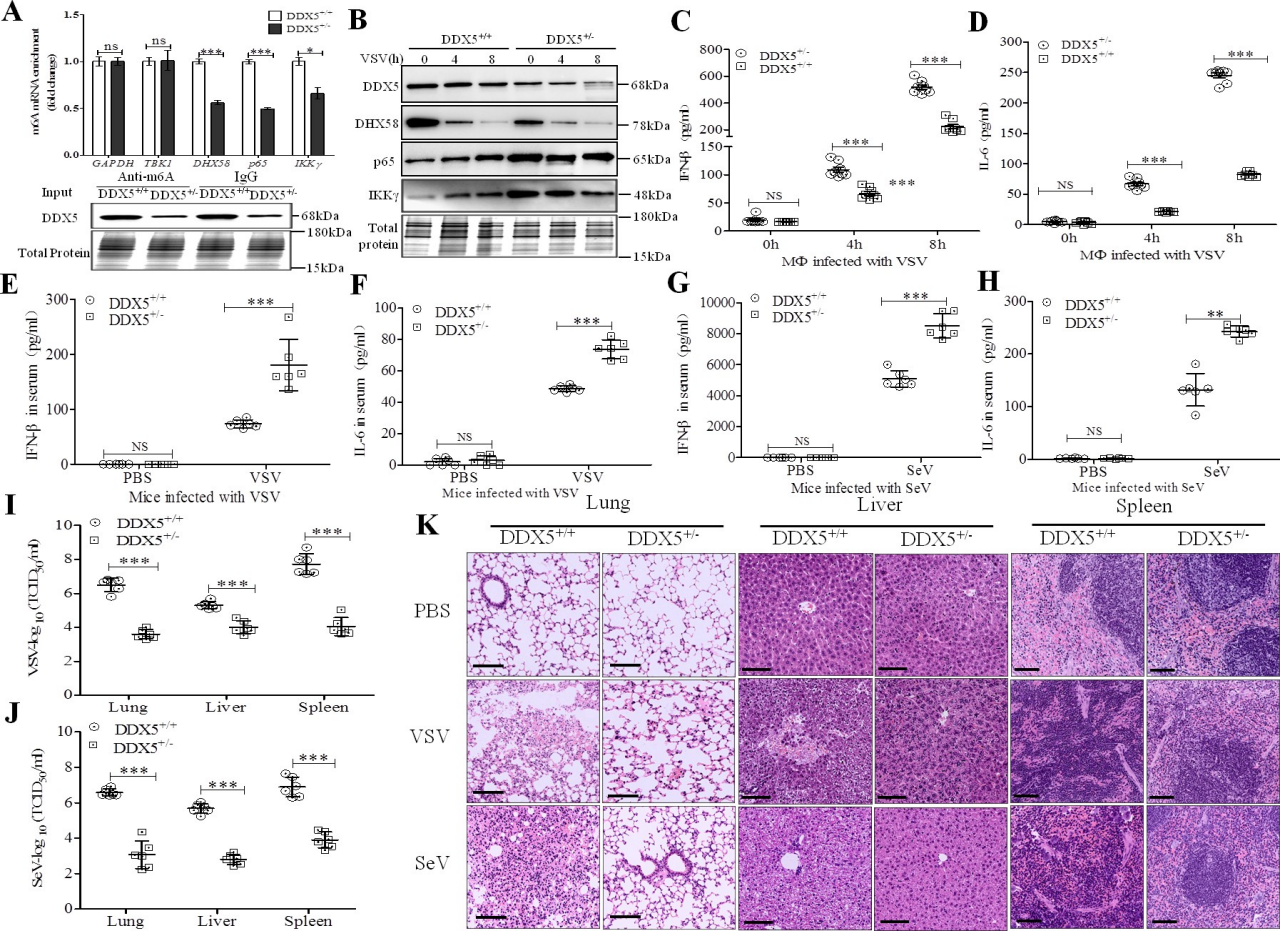
DDX5 negatively regulates innate immunity in vivo. **(A):** m6A methylation of transcripts was detected in DDX5^+/+^ or DDX5^+/-^primary mouse macrophages infected for 8 h with VSV (MOI = 10). After extracting total RNA and purifying mRNA, mRNA was used to perform m6A qRT-PCR by incubating with anti-rabbit m6A or anti-rabbit IgG-conjugated dynabeads for 4 h. RNA was isolated and subjected to qRT-PCR using GAPDH, TBK1, DHX58, IKKγ, and p65 primers. Results are presented relative to those obtained in the control group, and the expression of DDX5 was analyzed bywestern blotting. **(B):** Immunoblot analysis of DDX5, DHX58, p65, and IKKγ in lysates of DDX5^+/+^ or DDX5^+/-^mouse macrophages infected for 0, 4, and 8 h with VSV (MOI = 10). **(C, D):** ELISA of IFN-β (C) and IL-6 (D) in cell supernatants ofDDX5^+/+^ or DDX5^+/-^mouse macrophages infected for 0, 4, and 8 h with VSV (MOI = 10). **(E, F):** ELISA of IFN-β (E) and IL-6 (F) in serum after DDX5^+/+^ or DDX5^+/-^ mice were intraperitoneally injected with PBS or VSV (5×10^8^ plaque-forming units/g body weight) for 8h (n = 6). **(G, H):** ELISA of IFN-β (G) and IL-6 (H) in serum after DDX5^+/+^ or DDX5^+/-^ mice were intraperitoneally injected with PBS or SeV (1×10^8^ plaque-forming units/g body weight) for 8h (n = 6). **(I, J):** The TCID_50_ dose of VSV (I) or SeV (J) was measured in lungs, liver, and spleen of DDX5^+/+^ or DDX5^+/-^ mice. **(K):** Pathological lesions in lungs, liver, and spleen of DDX5^+/+^ or DDX5^+/-^ mice observed by hematoxylin-eosin staining with intraperitoneal injection of PBS, VSV (5×10^8^ plaque-forming units/g body weight) or SeV (1×10^8^ plaque-forming units/g body weight) for 12h. Scale bars, 100 μm. All data are presented as mean ± SEM of biologically independent samples. *n* = number of biological replicates. Data are representative of three independent experiments. NS, no significant difference. **p<0.01, ***p<0.001 (Student’s *t*-test).

To further investigate the role of DDX5 in vivo, we prepared DDX5 knockout mice by targeting exons 3–8 of the genomic DDX5 locus using CRISPR/Cas9-mediated genome engineering. We found that Ddx5^-/-^ mice died during the embryonic stage (date not shown), whereas a previous study showed that homozygous Ddx5^-/-^ mice with embryonic lethality or infertility displayed blood vessel malformations [41,42], suggesting a homozygous loss of function. However, DDX5^+/-^ progeny have a similar body weight to that of wild-type littermates, and they reached adulthood (S7A Fig); there was no difference in the organ coefficient (organ-body rate) between DDX5^+/-^ mice and wild-type littermates (S7B Fig). To evaluate the m6A methylation and expression of DHX58, p65, and IKKγ, we isolated peritoneal macrophages from DDX5^+/-^mice and wild-type littermates and infected them with VSV. As shown in Fig 8A, we found that the m6A methylation of DHX58, p65, and IKKγ transcripts was significantly decreased in DDX5^+/-^ primary mouse macrophages, while no change occurred in the methylation of transcripts (GAPDH and TBK1) not targeted to DDX5. Moreover, at 4 and 8 hpi, as shown in Fig 8B, the expression of DHX58 was decreased in DDX5^+/-^ mice compared with that of wild-type littermates, while p65 and IKKγ were obviously increased in DDX5^+/-^ mice compared with those of wild-type littermates, indicating that DDX5 regulated m6A methylation of transcript to activate the negative DHX58 pathway while inhibiting the positive p65 pathways after infection with VSV in innate immunity. Meanwhile, IFN-β and IL-6 showed significantly higher secretion in the culture supernatant of DDX5^+/-^ mouse peritoneal macrophages than that of wild-type littermates (Fig 8C and 8D). Moreover, we measured a significant increase in IFN-β and IL-6 production in DDX5^+/-^ mice compared to that of wild-type littermates infected with VSV (Fig 8E and 8F) or SeV (Fig 8G and 8H), which demonstrated that DDX5 negatively regulates innate immunity in vivo. To further observe the viral infection-mediated pathology, we measured the viral replication and conducted histopathological analysis after challenge with VSV or SeV (Fig 8I and 8J). Results showed that the viral titers in pathological tissue (lungs, liver, and spleen) of DDX5^+/-^ mice were obviously significantly lower than those of wild-type littermates (Fig 8I, 8J, and 8K), whereas less interstitial pneumonitis in the lungs, less damage in the liver, and fewer lesions in the spleen were observed in DDX5^+/−^ mice than in those of their wild-type littermates after viral challenge. Altogether, these results showed that DDX5 promotes viral replication and tissue lesions through negatively regulating innate immunity in vivo.

## Discussion

In this study, we clearly demonstrated that DDX5 acts as a negative antiviral regulator that manipulates N^6^-methyladenosine of transcripts in innate immunity. Furthermore, DDX5 could significantly inhibit IFN-β and IL-6 production after infection with RNA virus. Therefore, DDX5 may be a valuable target for blocking viral immune evasion. Our findings are also in line with those of a recent study, which reported that DDX5 suppresses IFN-I antiviral innate immune response by interacting with PP2A-Cβ to deactivate IRF3 [43]. However, because DDX is a large family of conserved RNA-binding proteins, nearly all DDX family members have been demonstrated to regulate cellular metabolism and maintain immune homeostasis through regulating nucleic acid binding and to modulate nucleic acid interactions and RNA-protein complex remodeling in host cells, such as DDX58, MDA5, DHX58, DDX3X, DDX21, DDX19, DDX39A, and DDX46 [2,13,14,15,16,44–51]. In fact, DDX5 has been demonstrated to regulate transcription in order to play its roles in tumorigenesis, proliferation, differentiation, and metastasis pathways [30,46]. Therefore, we hypothesized that DDX5 may operate in regulating transcription to manipulate innate immune response. We found that METTL3 was the major targeted protein in VSV-infected MEFs or macrophages. Coincidentally, the interaction of DDX5 and METTL3 has also been found in macrophage lipid uptake [52], which indicated that METTL3 is the pivotal target of DDX5 in cellular development and viral infection. Previous studies showed that METTL3 was the primary component of the writer complex of RNA m6A modification and is involved in regulating mRNA translation and other biological processes through epi-transcriptome modulation [53]. METTL3 not only mediates m6A modification to increase translation of immune transcripts to physiologically promote DC activation and DC-based T cells [54], but also serves as a negative regulator of the interferon response by dictating the fast turnover of interferon mRNAs and consequently facilitating viral propagation [29]. This hinted that DDX5 inhibited innate immunity by interacting with METTL3 after infection with RNA virus. In our study, we found that DDX5 interacted with METTL3 in the nucleus of VSV-infected cells, and N-terminal-deleted METTL3 lost its capacity to localize within the nucleus and could not interact with DDX5. Moreover, DDX5 lacking its P68HR domain also could not localize within the nucleus nor interact with METTL3; thus, we confirmed that the N-terminal domain of METTL3 and the P68HR domain of DDX5 are required for the molecular interaction between DDX5 and METTL3.

Previously, it was shown that m6A in mRNAs and other RNA polymerase II-derived transcripts are primarily formed by the METTL3–METTL14 heterodimer [33,34,55], which hinted that DDX5 may participate in the formation of the m6A writer complex and regulate RNA m6A methylation activity. Numerous studies reported that the m6A writer complex comprises METTL3 and its adaptor METTL14, which stabilizes METTL3, and that the proteins act together to mediate m6A formation [56]. Beyond that, little is known about how the writer complex is regulated. In our study, we found that the interaction of DDX5 and METTL3 was enhanced by viral infection. DDX5, METTL3, and METTL14 co-localized and interacted in the nucleus of VSV-infected cells, and DDX5 could regulate the interaction between METTL3 and METTL14. Taken together, these data indicated that DDX5 participated in the formation of the m6A writer complex after viral infection. Thus, it is possible that DDX5 is a vital regulator of theMETTL3-METTL14 complex. To further confirm these results, we detected the m6A content in total RNA in DDX5-knockdown and DDX5-overexpressing cells. We found that DDX5 could effectively affect the RNA m6A level of both MEFs and macrophages, indicating that DDX5 was a vital regulator of the m6A writer complex and that it participated in the RNA m6A methylation of host cells. This finding suggested that DDX5 and METTL3 could be involved in tumorigenesis by regulating gene transcription in previous studies [30,57].

A previous study showed that m6A inhibited the IFN-I response by dictating the fast turnover rate of IFN-β mRNAs [29]. However, we found that METTL3 has more strategies for effectively suppressing innate immune response. METTL3 was recruited by DDX5, even both of METTL3 and DDX5 could promote R-loop formation to facilitate transcription [46,58]; we hypothesized that METTL3 may be manipulated by DDX5 to control the RNA m6A of DDX5-bound mRNAs in innate immunity. Then, we employed iCLIP-seq to screen mRNAs bound to DDX5 in VSV-infected MEFs. The total amount of mRNAs that bound to DDX5 was reduced after infection with VSV; however, nearly all mRNAs bound to DDX5 at a conserved GCUGCAG element, which is different from that of previously reported RNAs bound to DEAD box helicases [14,59,60], which indicated that the GCUGCAG element is a unique binding site between DDX5 and various RNAs. Studies have demonstrated that RNA m6A methylation plays an important role in controlling gene transcription and expression on different levels [23,61,62]. Our findings demonstrated that DDX5 is a vital regulator of the m6A writer; furthermore, DDX5 could bind to RNAs to control gene transcription and expression involved in innate immunity. Therefore, we acquired the different expression transcripts from the differential transcripts bound to DDX5 between VSV-infected MEFs and uninfected MEFs by combining iCLIP-seq and RNA-seq analyses. Then, we identified several differentially expressed transcripts with roles in innate immunity and selected three transcripts (DHX58, p65, and IKKγ). Contrary to the enrichment of DDX46-bound RNAs after viral infection in previous study [14], we showed that these three transcripts bound to DDX5 at a reduced level after cells were infected with VSV, while there was an induction in m6A modification of the transcripts mediated by METTL3, which was also regulated by DDX5, indicating that DDX5 was associated with METTL3-mediated m6A modification of the transcripts and it may be a key mediator between m6A writer and the targeted transcripts. Studies have shown that m6A modification can wipe off or add to the nucleus or cytoplasm, depending on the function and location of the mRNAs [63]. Generally, about 25% mRNAs are m6A modified to play roles in immune response, biological metabolism, and differentiation, and the RNAs are in a dynamic equilibrium between m6A modified and non-modified states [64]. The m6A modification may enhance mRNA export from the nucleus through its binding to reader proteins [38], and blocking m6A modification results in delayed nuclear export of mature mRNA [22,65], while promoting the m6A modification results in rapid cytoplasmic appearance of mRNA [66]. In our study, our study showed that depletion of DDX5 caused m6A modified transcripts (p65, IKKγ and DHX58) were increasing in the nucleus while decreased in the cytoplasm, which resulted in the m6A modified transcripts could not be recognized by the reader proteins to suffer from degradation, all these would lead to a increasing expression of mature mRNA and proteins in the cytoplasm. In fact, we found that DDX5 could impel transcript export from the nucleus, and expression of DHX58 was promoted while the expression of transcript p65 and IKKγ were decreased; this mainly occurs because the m6A methylation of mRNAs promotes nuclear export and allows for increased RNA decay to occur in a m6A reader YTHDF2-dependent manner [22,37]. Indeed, we found that the remaining mRNAs of antiviral transcription were significantly increased in DDX5-knockout MEFs and were significantly reduced in DDX5-overexpressed MEFs, indicating that mRNA decay was involved in the regulation of innate immunity. RNA decay has been reported to play roles in several biological processes of mRNA m6A methylation, and the m6A reader YTHDF2 was the critical protein that mediated degradation of mRNAs by recognizing m6A methylation [67]. In our study, the transcripts (p65 and IKKγ) were significantly increased in DDX5-overexpressing MEFs when YTHDF2 was silenced, and binding of transcripts (p65 and IKKγ) to YTHDF2 was regulated by DDX5, but no effects were observed on transcripts of DHX58 (also named as LGP2), which negatively regulate innate immune signaling by recognizing viral dsRNA to inhibit RIG-I-and MDA5-mediated TBK1 and p65 pathways [47,68]. This result suggested that YTHDF2 could mediate the mRNA decay of transcripts (p65 and IKKγ) rather than that of the negative regulator DHX58, which indicated that regulation of transcripts by DDX5 may be dependent on the m6A mRNA destiny (degradation or translation) [67]. To confirm these results, we detected IFN-β and IL-6 production and VSV replication in YTHDF2-silenced cells along with DDX5-overexpressed MEFs. Compared with the results of DDX5-overexpressed MEFs, IFN-β and IL-6 production were significantly increased while the replication of VSV was significantly decreased in YTHDF2-silenced cells along with MEFs expressing DDX5, which indicated that DDX5 promoted RNA decay of antiviral transcripts in a YTHDF2-dependent manner. Therefore, our results demonstrate how viral infection can lead to altered regulation of the m6A modification of antiviral transcripts, thus promoting virulence.

Finally, we found that DDX5 inhibited the expression of phosphorylated TBK1, IKKγ, p65, and IRF7, and DDX5 also blocked the nuclear translocation of p65 and IRF7, which indicated that DDX5 inhibited the innate immunity by blocking the DHX58-mediated TBK1 and p65 pathways. Moreover, verification of these results using DDX5 knockout mice infected with VSV and SeV demonstrated that DDX5 was a negative antiviral regulator that manipulates N^6^-methyladenosine of transcripts in RNA virus-mediated innate immunity.

**Fig 9:**
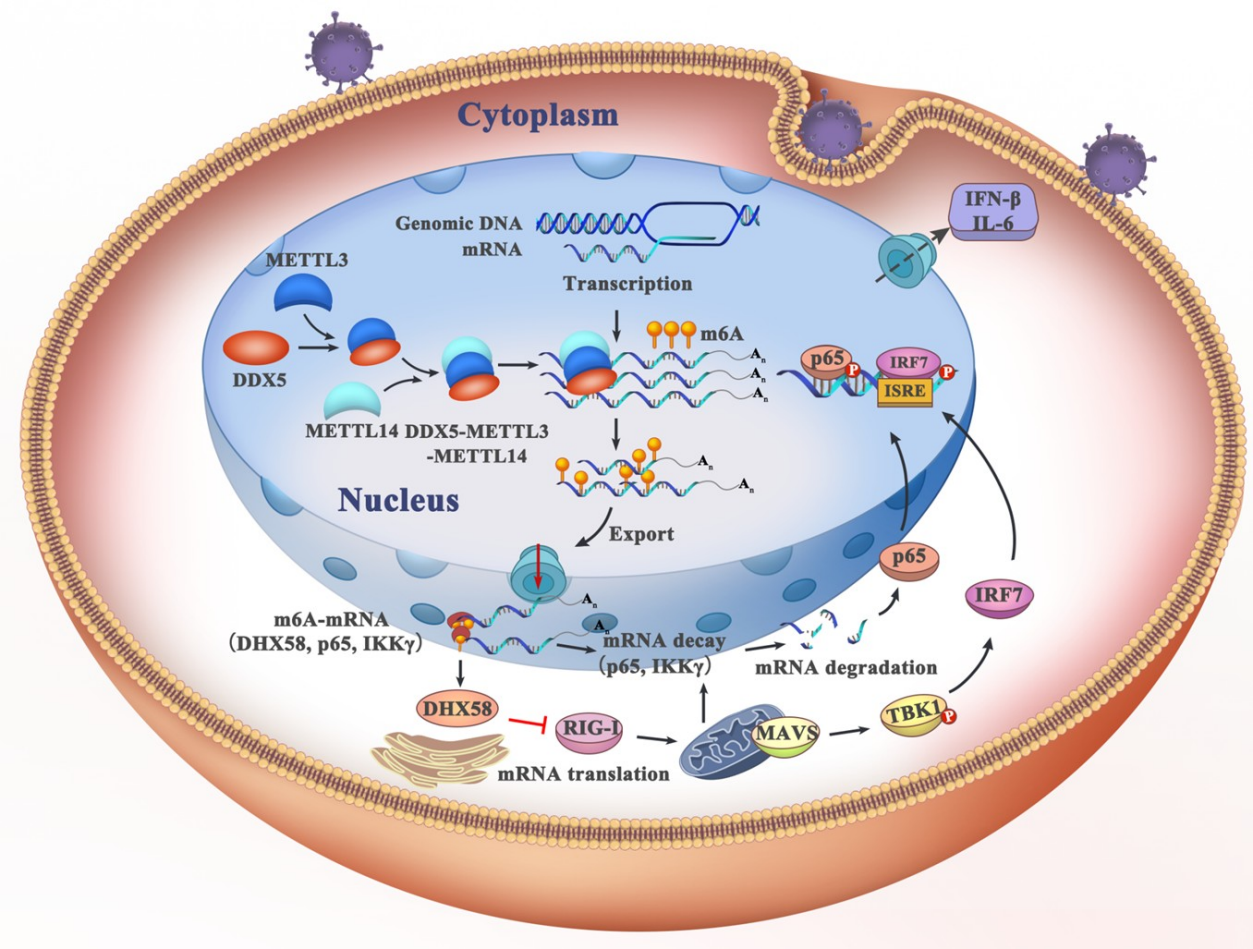
Working model for the mechanism through which RNA helicase DDX5 inhibits antiviral innate immunity by promoting m6A-methylated antiviral transcripts. DDX5 was aggregated in the nucleus of virus infected cells, which interacted with METTL3 and regulated the activity of the m6A ‘writer’ complex METTL3/METTL14. Then, the m6A ‘writer’ complex increased m6A modification of DHX58, p65, and IKKγ transcripts and sequentially exported them from nucleus to the cytoplasm, resulting in transcription of DHX58, which was also enhanced while the transcripts IKKγ and p65 were recognized by the m6A reader protein and were degraded in a YTHDF2-dependent manner, which finally resulted in the inhibition ofTBK1 and p65 mediated antiviral pathways.

In summary, we generated a model to elaborate on the mechanism of DDX5 in innate immunity. As shown in Fig 9, we found that DDX5 was aggregated in the nuclei of virus-infected cells, which regulated the activity of the m6A writer complex by interacting with METTL3. That DDX5 bound to antiviral transcripts underwent increased m6A modification and was exported from the nucleus to the cytoplasm; then, the transcripts IKKγ and p65 were recognized by the m6A reader protein and were degraded in a YTHDF2-dependent manner. Transcription of DHX58 was also enhanced, which finally resulted in the inhibition of the TBK1- and p65-mediated antiviral pathways. Our findings not only imply that DDX5 maybe turning the immune response off to maintain balance in the innate immune system, but also provide a novel target for developing antiviral therapeutics.

## Materials and methods

### Ethics statement

C57BL6/J specific-pathogen-free (SPF) mice (6–8 weeks old) and SPF chicken embryos(10 days old) were purchased from Beijing Vital River Laboratory Animal Technology Co., Ltd. (Beijing, China). All animal care procedures and experiments were approved by the Beijing Academy of Agricultural and Forestry Sciences Animal Care and Use Committee guidelines (ID: SYXK [Jing] 2017–0039), which were approved by the animal welfare committee of Beijing Academy of Agricultural and Forestry Sciences (15 December 2017).

### Cells and virus

MEFs (ATCC NIH/3T3) were purchased from the American Type Culture Collection (Manassas, VA, USA), and mouse macrophages (J774A.1) and baby hamster kidney (BHK) cells were purchased from the National Infrastructure of Cell Line Resource (Beijing, China). The MEFs and macrophages were maintained in Dulbecco’s modified Eagle’s medium with 10% fetal bovine serum (FBS). VSV and GFP-labeled VSV were proliferated and amplified in BHK cells. SeV was proliferated and amplified in 10-day-old SPF chicken embryos.

### Antibodies and reagents

All restriction enzymes were purchased from New England Biolabs (Ipswich, MA, USA). Rabbit DDX5 polyclonal antibody (ab21696), rabbit DDX5 monoclonal antibody (ab126730), mouse DDX5 monoclonal antibody (ab53216), rabbit METTL3 monoclonal antibody (ab195352), mouse METTL14 monoclonal antibody (ab220030), rabbit IKKγ monoclonal antibody (ab178872), mouse p65 monoclonal antibody (ab32536), Rabbit anti-VSV-G antibody (ab1874) and rabbit phospho-p65 (S276) monoclonal antibody (ab183559) were obtained from Abcam (Cambridge, MA, USA). Rabbit phospho-IRF-7 (Ser437/438) (D6M2I) monoclonal antibody (MouseSpecific) (#24129) was obtained from Cell Signaling Technology (Danvers, MA, USA). Phospho-NAK/TBK1-S172 rabbit monoclonal antibody (AP1026), DHX58rabbit polyclonal antibody (A8257), NAK/TBK1 (N-term) rabbit monoclonal antibody (A3458), phospho-IKKγ-S376 rabbit polyclonal antibody (AP1111), rabbit IRF7 polyclonal antibody (A0159), mouse β-actin monoclonal antibody (AC004), and rabbit GAPDH monoclonal antibody (A19056) were obtained from ABclonal Inc. (Wuhan, China). Anti-Flag mouse monoclonal antibody, anti-Myc rabbit monoclonal antibody, mouse METTL3 monoclonal antibody (OTI1B7) (MA5-27527), Alexa Fluor 555 Phalloidin (A34055), Alexa Fluor 546 F(ab′)_2_ fragment of goat anti-rabbit IgG (H+L) (A11071), Alexa Fluor 488 F(ab′)_2_ fragment of goat anti-mouse IgG (H+L) (A11017), goat anti-rabbit IgG (H+L) secondary antibody, DyLight 405 (35551), goat anti-mouse IgG (H+L) highly cross-adsorbed secondary antibody, Alexa Fluor 647 (A-21236) Lipofectamine 3000 DNA transfection reagents (L3000015), Lipofectamine RNAiMAX transfection reagent (13778150), and Pierce protein A/G magnetic beads (88803 and 26162) were purchased from Invitrogen (Thermo Fisher, Waltham, MA, USA). Poly (I: C) was obtained from InvivoGen (Toulouse, France). Poly(I:C) was obtained from InvivoGen (Hong Kong, China). Mouse DDX5 siRNA, METTL3 siRNA, and negative control siRNA were designed and synthesized by GenePharma (Shanghai, China). Adenovirus ADV1 vector was obtained from GenePharma (Shanghai, China). PWPXL vector was stored in our lab. An RNeasy Mini Kit was obtained from QIAGEN (Dusseldorf, Germany). The pEGF-N1 and pCMV-Myc-tag plasmids (Novagen, Darmstadt, Germany) were used for DNA manipulations. All other chemical reagents used in the study were of analytical grade.

### RNA interference and vectors

The mouse DDX5 (ID: 13207) gene was sub-cloned into the pEGF-N1, p 3×FLAG-CMV-10, and pCMV-Myc expression vectors. The plasmids were then transformed into *E*. *coli* T1 cells, which were then propagated in Luria–Bertani media, and progeny plasmids from *E*. *coli* were extracted using a plasmid kit (Qiagen, Dusseldorf, Germany). The truncated DDX5 mutants were designed and sub-cloned into the pCMV-Myc vector, introduced into *E*. *coli* T1 cells, and extracted using a plasmid kit (Qiagen, Dusseldorf, Germany). The mouse METTL3 (ID:56335) gene and its truncations were sub-cloned into p3×FLAG-CMV-10 (Novagen, Darmstadt, Germany) and extracted using a plasmid kit as per the manufacturer’s instructions.

MEFs were transfected with siRNA (final concentration, 5nM) using Lipofectamine RNAiMAX according to the manufacturer’s instructions. All siRNAs were obtained from GenePharma (Suzhou, China). The efficient siRNAs of DDX5 and METTL3 were constructed into the adenovirus ADV1 vector, and the ADV1-siDDX5 or ADV1-siMETTL3 vectors coupled with a shuttle vector were transfected into 293A cells with RNAi-Mate reagent (GenePharma, G04001) for 7–15 days. Then, the recombinant ADV1-siDDX5 and ADV1-siMETTL3 were recovered. The negative control siRNA was recombined into the ADV1 vector as an adenovirus control. All recovered adenovirus was stored at -80°C. The DDX5 gene was cloned into the lentiviral PWPXL vector, three vectors (PWPXL, PSPA, and PMD2 vectors) were co-transfected into 293A cells with FnGENE HD reagent (Promega, Madison, WI, USA) for 2 days, and then the recombinant lentiviral DDX5 was recovered and stored at -80°C.

### Cell treatments, transfection, and ELISA

The MEFs were transfected with siRNA using Lipofectamine RNAiMAX reagents to silence the targeted genes; otherwise, the MEFs were transfected with plasmids using Lipofectamine 3000 reagent to express target proteins. HEK293T cells were co-transfected with several plasmids using Lipofectamine 3000 reagent to detect protein interactions. Mouse macrophages were infected with DDX5 lentiviruses to express DDX5 in the cells, or they were infected with siRNA adenovirus to silence the targeted gene. For ELISA, the supernatant from DDX5/METTL3-silenced or DDX5/METTL3-expressing MEFs was collected at 0–24hpi with VSV (MOI = 10) or SeV (MOI = 5), and the supernatants were analyzed by the LumiKine Xpress mIFN-β 2.0 ELISA Kit (InvivoGen, Hong Kong, China), mouse IL-6 ELISA Kit, and mouse TNF-α ELISA Kit (Solarbio, Beijing, China).The m6A content in total RNA was detected and calculated with the m6A RNA Methylation Assay Kit (Colorimetric) (Abcam, ab185912).

### qRT-PCR

MEFs or macrophages were transfected with siRNA or plasmid(s). At 48 h post-transfection, total RNA was extracted via the RNeasy Mini Kit, and cDNA was synthesized using the Super Script IV First-Strand Synthesis System (Thermo Fisher, Waltham, MA, USA). Quantitative real-time PCR was performed using the iTaq Universal SYBR Green Supermix (Bio-Rad, Hercules, CA, USA) on a Real-Time System (Bio-Rad, Hercules, CA, USA) with the following primers (forward, reverse): mouse IFN-β (5′-CAGCTCCAAGAAAGGACGAAC-3′, 5′-GGCAGTGTAACTCTTCTGCAT-3′), mouse IL6 (5′-CACGGCCTTCCCTACTTCAC-3′, 5′-TGCAAGTGCATCATCGTTGT-3′), mouse p65 (5′-ATCATCGAACAGCCGAAGCA-3′, 5′-GTTCCTGGTCCTGTGTAGCC-3′), mouse IKKγ (5′-TCAAGAGCTCCGAGACGCTA-3′, 5′-AAGGCCTGTTCCCTCTGACT-3′), mouse DHX58 (5′-TACGACAGAGAACGCACCAC-3′, 5′-CAGCATCTCCAACTTCGGGT-3′), mouse RNU6 (5′-GTGCTCGCTTCGGCAGCA-3′, 5′-AATATGGAACGCTTCACGAAT-3′), mouse GAPDH (5′-AGGTCGGTGTGAACGGATTTG-3′, 5′-TGTAGACCATGTAGTTGAGGTCA-3′) and mouse TBK1(5′-CGGAGACCCGGCTGGTATAA-3′, 5′-ATCCACTGGACGAAGGAAGC-3′).

### Immunoblot analysis

Cells were lysed by RIPA buffer, and lysates were centrifuged at 12000 rpm/min for 10 min at 4°C and then measured using a BCA Protein Assay Kit (Thermo Fisher, Waltham, MA, USA). Samples were separated by 12% SDS-PAGE gel (Bio-Rad, Hercules, CA, USA), blotted onto polyvinylidene fluoride (PVDF) membranes, and immunoblotted with primary antibodies: anti-mouse DDX5 (1:2000), anti-rabbit DDX5 (1:10000), anti-METTL3 (1:1000), anti-METTL14 (1:750), anti-IRF7 (1:750), anti-β-actin (1:20000), anti-phospho-IKKγ (1:1000), anti-phospho-TBK1 (1:1000), anti-p65 (1:1000), anti-VSV-G (1:1000), anti-phospho-p65 (1:2000), anti-DHX58 (1:1000), anti-TBK1(1:1000), anti-TBK1(1:1000), Anti-IKKγ (1:1000), anti-phospho-IRF7 (1:1000), anti-mouse Myc (1:1000), anti-rabbit Myc (1:1000), and Anti-mouse Flag (1:1000). Secondary antibodies used were as follows: goat anti-rabbit IgG (H+L) secondary antibody, horseradish peroxidase (HRP) (1:3500), goat anti-mouse IgG (H+L) secondary antibody, HRP (1:10000), IRDye 800CW goat anti-rabbit (1:10000), and IRDye 680CW goat anti-rabbit (1:10000). Reactive bands were detected by a Imaging System (Bio-Rad, Hercules, CA, USA).

### RNA decay assays

To measure the stability of RNA transcripts in DDX5 knockout cells during VSV infection, we performed the RNA decay assay in MEFs by adding actinomycin D (Sigma Aldrich, St. Louis, MO, USA) at a final concentration of 5μM, the assay was performed according to a previous study [29,39]. At each time point, three independent wells each of DDX5-depleted cells and control cells were collected. Meanwhile, to measure the stability of DDX5-bound RNA, which is related to the YTHDF2 pathway, we calculated the RNA decay rate in MEFs with YTHDF2 knockdown and DDX5 expression, and the RNA decay rates were calculated as follows: all pretreated MEFs were added to actinomycin D (5μM); then, we normalized the number of reads for each transcript to follow the global degradation rate at each time point based on the median degradation level in mammalian cells. The decay rate for each transcript was calculated as the slope of the linear regression of the log of the normalized number of reads as a function of time.

### Pull-down assay, co-IP, and mass spectrometry

For the DDX5 pull-down assay, MEFs or macrophages were seeded and cultured on 60-mm dishes for 24 h, infected with VSV for 6h, and were lysed with NP40 lysis buffer containing protease inhibitor cocktail (Roche, Basel, Switzerland), and the pull-down assay was performed according to a previous study [13]. The specific protein bands were separated from the pull-down assay and sequenced by Q-TOF PREMIER technology (BGI, Beijing, China). Co-IP was performed to confirm the interaction between DDX5 and METTL3 in 293T cells. The 293T cells seeded on 60-mm dishes were transfected with 12 mg Myc-DDX5 (or Myc-DDX5 mutants) and Flag-METTL3 (or Flag-METTL3 mutants) expression plasmids. At 24h post-transfection, cells were lysed with NP40 cell lysis buffer containing protease inhibitor cocktail (Roche, Basel, Switzerland). Lysates were centrifuged at 12,000 rpm for 10 min at 4°Cand were precipitated with a mouse anti-Myc antibody or mouse anti-Flag antibody in conjunction with protein G/A-magnetic beads (Thermo Fisher, Waltham, MA, USA). The beads were washed with cold phosphate-buffered saline (PBS) four times and eluted with sodium dodecyl sulfate (SDS) loading buffer (TransGen, Beijing, China) by boiling for 10 min. Proteins isolated from the beads and cell lysates were separated by SDS-polyacrylamide gel electrophoresis and analyzed by western blotting using anti-Myc and anti-DDX5 antibodies. For Co-IP to confirm the interaction between endogenous DDX5 and METTL3, MEFs were seeded on 60-mm dishes and infected with VSV for 6–8h, and Poly (I: C)-treated MEFs were used as the positive control. Then, cells were lysed with cell lysis buffer NP40, the lysates were precipitated with a rabbit DDX5antibody in conjunction with protein G/A-magnetic beads (Thermo Fisher, Waltham, MA, USA), and Co-IP was operated as described above. For Co-IP ofMETTL3 and METTL14, MEFs were seeded on 60-mm dishes and transfected with different doses of siRNA or enhanced green fluorescent protein (EGFP) fused DDX5 expression plasmids, and then the cells were infected with VSV for 6–8h and lysed with lysis buffer NP40. Lysates were precipitated with a rabbit METTL3 antibody in conjunction with protein G/A-magnetic beads (Thermo Fisher, Waltham, MA, USA), and co-IP was performed as described above.

### iCLIP assay and data analysis

The iCLIP assay was conducted according to the previously reported protocol [68,69] with the following modifications: the MEFs were infected with VSV for 6 h or remained uninfected. Then, the cells were crosslinked with 0.4 J/cm^2^ of 254 nm UV light in a XL-1000 UV Crosslinkers XL-1500 (Spectronics, Westbury, NY, USA). The cells were lysed by NP40 containing a protease inhibitor cocktail, and lysates were pre-treated with 1/500 diluted RNaseI (Ambion, Austin, TX, USA; AM2295) and 1/500 diluted DNase (Thermo Fisher, AM2238). The lysates were incubated with anti-rabbit DDX5 or anti-rabbit IgG-conjugated protein G dynabeads (Thermo Fisher, 10004D) overnight at 4°C. After IP, a fraction of beads was used to label biotin with the Pierce RNA 3′end biotinylation kit (Thermo Fisher, 20160), beads with or without labeled biotin were separated on a NuPAGE 4–12% Bis-Tris Gel (Invitrogen, Carlsbad, CA, USA; NP0335BOX), and the protein–RNA complexes were transferred to a PVDF membrane. Biotin-labeled RNA was detected and visualized according to the instructions of the chemiluminescent nuclei acid detection module (Thermo Fisher, 89880), the biotin-unlabeled RNA was acquired according to the biotin-labeled protein–RNA complex blotting, and mRNAs were purified with the Dynabeads mRNA Purification Kit (Invitrogen, 61006). Then, mRNAs were subjected to small-RNA library construction with Collibri Stranded RNA Library Prep Kit for Illumina Systems with Human/Mouse/Rat rRNA Depletion Kit (Thermo Fisher, A39003096), the libraries were sequenced by standard Illumina protocols with PE150 (Allwegene, Beijing, China), and bioinformatics analyses were performed according to the requirements as described previously.

### RNA-binding protein IP (RIP) RT-qPCR and m6A qRT-PCR

DDX5-bound transcripts were identified by RIP qRT-PCR as previous study described [14,70]. MEFs were infected with VSV for 6 h or remained uninfected, and the cells were crosslinked with 0.4 J/cm^2^ of 254 nm UV light in Crosslinkers. Then, cells were lysed by NP40 buffer containing a protease inhibitor cocktail and RNase inhibitor, and the lysates were incubated with anti-rabbit DDX5, or anti-rabbit IgG-conjugated dynabeads overnight at 4°C. RNAs were isolated and purified from the solution with phenol-chloroform, and cDNA was subjected to qRT-PCR. To analyze the DDX5-binding ability, relative enrichment was normalized to input and compared to anti-IgG control, as previously described [14,71]. METTL3 RIP RT-qPCR was performed as follows: MEFs were transfected with DDX5 siRNA (5 nM) or siNC (5 nM) for 48 h, or transfected with DDX5-expressing plasmids or control vector for 24 h. Then, the cells were infected with VSV (MOI = 10) for 6 h. Next, the cells were lysed and subjected to RIP-qPCR with anti-rabbit METTL3 or anti-rabbit IgG-conjugated dynabeads, as described above. YTHDF2 RIP-qPCR was performed with DDX5 KO or overexpressing MEFs, which were infected with VSV (MOI = 10) for 6 h, and the cells were lysed and subjected to RIP-qPCR with anti-rabbit YTHDF2 or anti-rabbit IgG-conjugated dynabeads, as described above. The m6A qRT-PCR was operated according to a previous study [72] with some modification. MEFs were infected with VSV for 6 h or remained uninfected; then, the cells were collected to extract the total RNA, mRNA was purified, ribosomal RNA was removed, the purified mRNA was fragmented with fragmentation buffer (Thermo Fisher, AM8740), and then the mRNA was incubated with anti-rabbit m6A or anti-rabbit IgG-conjugated dynabeads for 4 h at 4°C. RNAs were isolated from the solution with phenol-chloroform, and cDNA was subjected to qRT-PCR. The nucleo-cytoplasmic fractionation of RNA in DDX5/METTL3-silenced or DDX5/METTL3-overexpressed MEFs were performed as described by a previous study [14,15], and the purified cytoplasmic and nuclear RNAs were subjected to m6AqRT-PCR to detect m6A DHX58, m6A IKKγ, and m6A p65, with GAPDH as the cytoplasmic control and RNU6 as the nuclear control. The m6A qRT-PCR was employed for primary peritoneal macrophages from the DDX5^+/-^ mice and wild-type DDX5^+/+^littermates as follows: the primary peritoneal macrophages were infected with VSV (MOI = 10) for 4 h, and then the cells were collected to extract RNA for m6A RT-qPCR, as described above and in previous studies [72,73]. The relative enrichment of each transcript was determined through quantitative real-time PCR and calculated using the ratio of RNA abundances of IP/input, and the relative enrichment fold change was calculated using the cycle threshold(CT) ^2(-ΔCT)^ method [73,74]. Briefly, the relative enrichment was first normalized to input and then analyzed via normalization to the data from the control sample immunoprecipitated with the targeted antibody. All samples were analyzed in triplicate for qPCR.

### RNA-seq and data analysis

MEFs were infected with VSV for 6 h or remained uninfected, total RNA was isolated using TRIZOL Reagent (Invitrogen, 15596026), and the cDNA library was constructed and sequenced with theIlluminaHiseq4000 system (Illumina Inc., San Diego, CA, USA). Transcript expression was quantified according to the fragments per kilobase of exon model per million mapped reads (FPKM) value (FPKM>1), the Venn diagram of genes was prepared based on differences between VSV-infected and uninfected MEFs, and the Venn diagram of transcripts bound to DDX5 was also prepared according to differences determined by iCLIP-seq and RNA-seq. Based on the differences in both expression and binding to DDX5 after VSV infection, we screened the DDX5-bound transcripts that were related to innate immunity. Then, we selected three transcripts that have been demonstrated to play a vital role in activating the innate immunity and made a heat map of the expression of the three transcripts according to the FPKM values. To analyze the characteristics of the three DDX5-bound transcripts in the genome, we drew the distribution of a single transcript in the genome using the IGV visualization tool for interactive exploration of genomic data sets.

### Depletion and generation of DDX5 knockout mice by CRISPR/Cas9 technology

For the depletion of DDX5 in MEFs, the pSpCas9n (D10A) plasmid coupled with specific DDX5 guide RNA were transfected into MEFs. After transfection, the MEFs with green fluorescence were sorted by a flow cytometer (BD FACSARIA II, Franklin, NJ, USA). Sorted cells were cultured with hygromycin B (Selleck, Houston, TX, USA; S2908) for 2–4 days, and then the clones were propagated from single cells. After several passages, DDX5 depletion was confirmed by western blot and DNA sequence analysis. The guide RNAs were as follows: DDX5-sgR-1: 5′-AAACAGGTTTAGCAGTCTATTGG-3′; DDX5-sgR-2: 5′-TGCGTGCTTGTGTAGTGACTAGG-3′.

DDX5-knockout mice were generated by Cyagen Biosciences Inc. (Shanghai, China) using CRISPR/Cas9 technology. Briefly, gRNA to the mouse DDX5 gene and Cas9 mRNA were co-injected into fertilized mouse eggs to generate targeted knockout offspring. F0 founder animals were identified by PCR followed by sequence analysis, which were bred with wild-type mice to test germline transmission and F1 animal generation. Founder mice were hybridized with wild-type C57BL6/J mice to produce the mice identified and used for experiments.

### Immunofluorescence analysis (IFA) and confocal imaging

The MEFs, macrophages, or 293T cells were seeded on coverslips in 12-well plates and cultured for 18–24 h. The cells were then infected with VSV or transfected with plasmids or siRNAs, The cells were fixed with 1% paraformaldehyde, permeabilized with 0.1% Triton X-100 for 30 min, and blocked with 5% FBS. To observe the co-localization of DDX5, METTL3, and METTL14 in cells, MEFs were transfected with EGF-DDX5 plasmid for 24h, and then the slides were incubated with anti-METTL3and anti-METTL14 antibodies for 1.5 h at room temperature. To assess the distribution of p65 or IRF7 in MEFs, slides were incubated with anti-p65 and anti-IRF7 antibodies for 1.5 h at room temperature. Sections were then incubated with Alexa Fluor 546 F(ab′)2 fragment of goat anti-rabbit IgG (H+L), Alexa Fluor 488 F(ab′)2 fragment of goat anti-mouse IgG (H+L), or goat anti-rabbit IgG (H+L) secondary antibody, DyLight 405. Nuclei were counterstained with4′,6-diamidino-2-phenylindole(DAPI), and cellular F-actin was stained with Alexa Fluor 555-conjugated phalloidin. Cells were then observed with a CLSM (Nikon, Tokyo, Japan).

### Immunofluorescence combined with RNA fluorescence *in situ* hybridization (FISH) assay

MEFs were infected with VSV (MOI = 10) for 6 h, fixed in 4% paraformaldehyde, and subjected to IF combined with FISH assay, as described previously [75,76]. The transcripts were labeled with the probes, and the DDX5 and METTL3 proteins were labeled with mouse METTL3 monoclonal antibody (Thermo Fisher, Waltham, MA, USA) and rabbit DDX5 monoclonal antibody (Abcam, Cambridge, MA, USA), followed by goat anti-rabbit IgG (H+L) secondary antibody, DyLight 405, and goat anti-mouse IgG (H+L) highly cross-adsorbed secondary antibody, Alexa Fluor 647 (Thermo Fisher, Waltham, MA, USA). Images were obtained using a CLSM (Nikon, Tokyo, Japan). Probes of *DHX58*, *p65*, and *IKK*γ with 5-biotin modification were designed by GenePharma (Suzhou, China), and the probes of RNA FISH negative control (NC) were provided by GenePharma (F44202). All probes were labeled with carboxyfluorescein (FAM) using a FISH SA-Biotin system (GenePharma, F12202/50). The sequences of probes of *DHX58* transcript were: 5′-AGGATGGGGTTTGGAAATGAAT-3′; 5′-CAAGATGGTGTTGTAGACGGTG-3′; 5′-GTGGATAAATAGCGCATCATTG-3′; 5′-AGGAAGGAGTACACACTCTGAC-3′; 5′-CATACAGTTGATGCAGAGAAGT-3′; those of probes of *p65* transcript were: 5′-GCATTTATAGCGGAATCGCCATG-3′; 5′-CGCACAGCAAGAAGATCTCATC-3′; 5′-ACAGAAGTTGAGTTTCGGGTAG-3′; 5′-AATGTGTACGCACTTCGGGAAG-3′; 5′-TAGAAGCTGGAGATGGAAGCAG-3′; and those of probes of *IKKγ* transcripts were: 5′-CAAATGAAAGGAGTGGTGAGCT-3′; 5′-TGCTTGGCAAAAACAAGAGCAC-3′; 5′-TGTTTATCCAAGAAGCGTGAAG-3′; 5′-ATAGGCAGAAGAAACCTCAGAG-3′; 5′-CAAGTGACGTCCATGAAGATAT-3′.

### Luciferase reporter gene assay

Luciferase activities were measured with a Dual-Luciferase Reporter (DLR) Assay System (Promega, Madison, WI, USA) according to the manufacturer’s instructions. Briefly, HEK293Tor MEFs were seeded into 24-well plates and were co-transfected with 500 ng luciferase reporter (IFNβ-Luc or IL6-Luc), 500 ng Myc-DDX5 plasmids (or control vector), and 20 ng pRL-TK plasmid. After 24 h post-transfection, the lysed samples were prepared, and luciferase activity was detected by the DLR Assay System (Promega, Madison, WI, USA). Data were normalized for transfection efficiency by dividing the Firefly luciferase activity by that of Rlla luciferase.

### Histopathological analysis

The lung, liver, and spleen of wild-type and DDX5^+/-^ mice infected with VSV or SeV were fixed in 4% paraformaldehyde overnight. Tissues were prepared in sections for histopathological analysis, and results were analyzed by light microscopy.

### Statistical analysis

All tests were performed in accordance with the biostatistical requirements. All the dates in this study were analyzed in triplicate. Statistical differences between the treated and control groups were determined and analyzed by analysis of variance using SPSS software, version 18.0 (SPSS, Chicago, IL, USA). Graphs were generated using GraphPad Prism 5.0. “ns” indicates no significant difference (P>0.05). *, **, and *** indicate statistically significant differences with values of P<0.05, P<0.01, and P<0.001, respectively.

## Supporting information

S1 Fig Expression of DDX5 in 3T3 MEF cells after transfected with DDX5 vector or siRNAs. **A**: 3T3 cells were cultured in 6-well plates and were transfected with siRNA negative control (siNC) or siDDX5 for 24h, then the cells were infected with GFP-VSV(MOI = 10) for 0, 4, 8h, and the expression of DDX5 was detected with western blot. **B**: 3T3 cells were cultured in 6-well plates and were transfected with pCMV myc vector (Con) or pCMV-DDX5 vector for 24h, then the cells were infected with GFP-VSV(MOI = 10) for 0, 2, 6, 8h, and the expression of myc-DDX5 was detected with western blot.
(TIF)

S2 Fig DDX5 pull down assay in MEF or mouse macrophages after infected with VSV. MEFs or macrophage(MФ) was seeded and cultured on 60-mm dishes for 24 h, then the cells were infected with VSV for 6h and were lysed with NP40 lysis buffer containing protease inhibitor cocktail (Roche), lysates were centrifuged at 12,000 rpm for 10 min and were precipitated with a Rabbit anti-DDX5 antibody or Rabbit IgG in conjunction with protein G/A-magnetic beads (Thermofisher, MA, USA). The beads were washed with cold PBS four times and eluted with SDS loading buffer (TransGen, Beijing, China) by boiling for 10 min. Proteins isolated from the beads and the cell lysates were separated by SDS-PAGE, then the PAGE gel resolution of immunoprecipitated DDX5 and its associated proteins from MEFs or macrophage(MФ) infected by VSV for the indicated time. Different bands were analyzed by MS. Arrow indicates the band of the METTL3 protein detected by MS.
(TIF)

S3 Fig Frequency of total peaks under substantial iCLIP-seq date. Frequency of total peaks under substantial iCLIP-seq date in MEF left uninfected or infected with VSV (MOI = 10). UTR, untranslated region; TSS, transcriptional start site.
(TIF)

S4 Fig The observation of co-location of transcripts/DDX5/METTL3 by FISH and CLSM. MEFs were infected with VSV (MoI = 10) for 6 h and fixed in 4% paraformaldehyde, the probes of trancripts were labeled with FAM using a FISH SA-Biotin system to suffer from IF combined with FISH assay. The transcripts were labeled with the probes, and the DDX5 protein and the METTL3 protein were labeled with mouse METTL3 monoclonal antibody and rabbit DDX5 monoclonal antibody following with goat anti-rabbit IgG (H+L) secondary antibody, DyLight 405 and goat anti-mouse IgG (H+L) highly cross-adsorbed secondary antibody, Alexa Fluor 647, then images were obtained with a CLSM. DDX5 was labeled with DyLight 405 fluorescent (Blue), METTL3 was labeled with Alexa Fluor 647 fluorescent (Red), and the transcripts were labeled with FAM-Probes (Green). A: RNA FISH negative control (NC); B: DHX58; C: IKKγ; D: p65. Scale bars, 20 μm.
(TIF)

S5 Fig The stability of GAPDH and TBK1 in DDX5-knockout or over expressed MEFs. GAPDH mRNA degradation in VSV infected DDX5-knockout MEFs(A) or overexpressed MEFs (B) treated with actinomycin D at indicated times (n = 3). TBK1 mRNA degradation in VSV infected DDX5-knockout MEFs(C) or overexpressed MEFs (D) treated with actinomycin D at indicated times (n = 3). Residual RNAs were normalized to 0 h.
(TIF)

S6 Fig The knockdown effects of DDX5 in MEFs. Knockdown effects of DDX5 were analyzed by immunoblot in MEFs transfected for 48 h with non-targeting control siRNA (NC) or YTHDF2-specific siRNA (siYTHDF2 (one of three siRNA constructs [si#1, si#1 and si#1]).
(TIF)

S7 Fig Quantification of organ-body rate in DDX5+/+and DDX5+/-mice. **A:** Quantification of DDX5^+/+^ or DDX5^+/-^ mice at postnatal 3 and 4 weeks (n = 6). **B**: Organ-body rate was quantified between DDX5^+/-^ mice and wild-type littermates (n = 6).
(TIF)

S1 Table RNA oligonucleotides used for the depletion of cell genes in this study. (DOCX)

S2 Table Primers used for gene cloning. (DOCX)
