# Supplementary figures and images for: The RNA helicase DDX5 promotes viral infection via regulating N6-methyladenosine levels on the DHX58 and NFκB transcripts to dampen antiviral innate immunity
Source: PLoS Pathog. 2021 Apr 28;17(4):e1009530. doi: 10.1371/journal.ppat.1009530 (PMC8081163; doi:10.1371/journal.ppat.1009530)

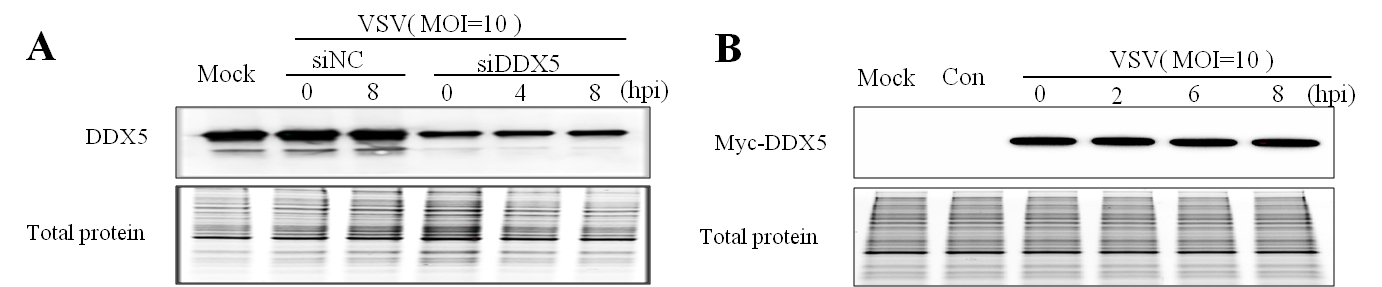

Supplement: S1 Fig — A: 3T3 cells were cultured in 6-well plates and were transfected with siRNA negative control (siNC) or siDDX5 for 24h, then the cells were infected with GFP-VSV(MOI = 10) for 0, 4, 8h, and the expression of DDX5 was detected with western blot. B: 3T3 cells were cultured in 6-well plates and were transfected with pCMV myc vector (Con) or pCMV-DDX5 vector for 24h, then the cells were infected with GFP-VSV(MOI = 10) for 0, 2, 6, 8h, and the expression of myc-DDX5 was detected with western blot. (TIF) [file ppat.1009530.s001.tif]

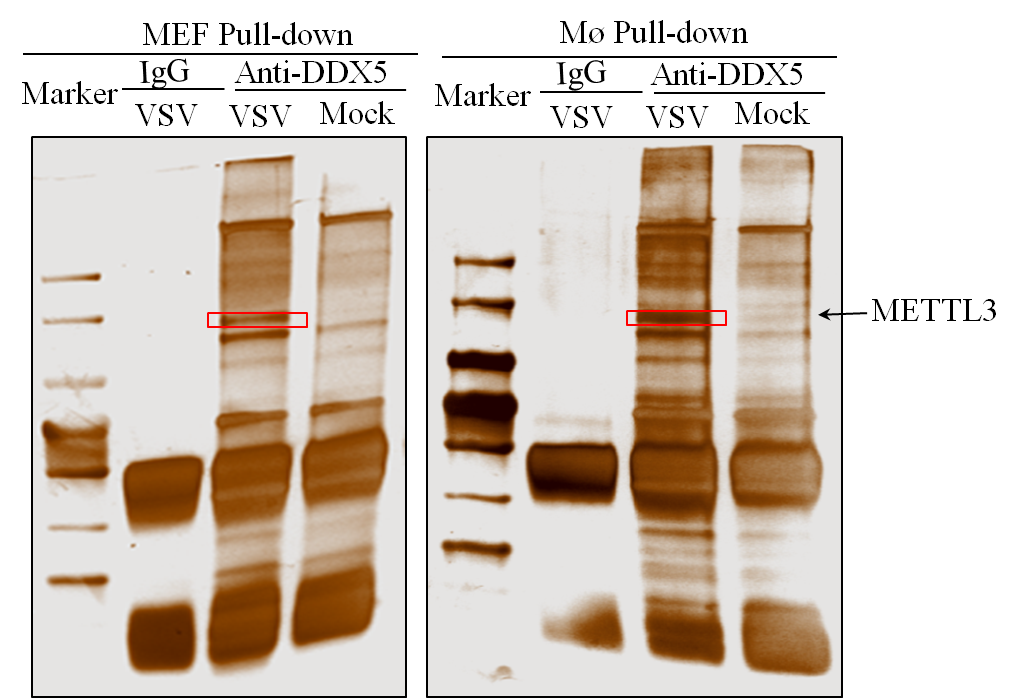

Supplement: S2 Fig — MEFs or macrophage(MФ) was seeded and cultured on 60-mm dishes for 24 h, then the cells were infected with VSV for 6h and were lysed with NP40 lysis buffer containing protease inhibitor cocktail (Roche), lysates were centrifuged at 12,000 rpm for 10 min and were precipitated with a Rabbit anti-DDX5 antibody or Rabbit IgG in conjunction with protein G/A-magnetic beads (Thermofisher, MA, USA). The beads were washed with cold PBS four times and eluted with SDS loading buffer (TransGen, Beijing, China) by boiling for 10 min. Proteins isolated from the beads and the cell lysates were separated by SDS-PAGE, then the PAGE gel resolution of immunoprecipitated DDX5 and its associated proteins from MEFs or macrophage(MФ) infected by VSV for the indicated time. Different bands were analyzed by MS. Arrow indicates the band of the METTL3 protein detected by MS. (TIF) [file ppat.1009530.s002.tif]

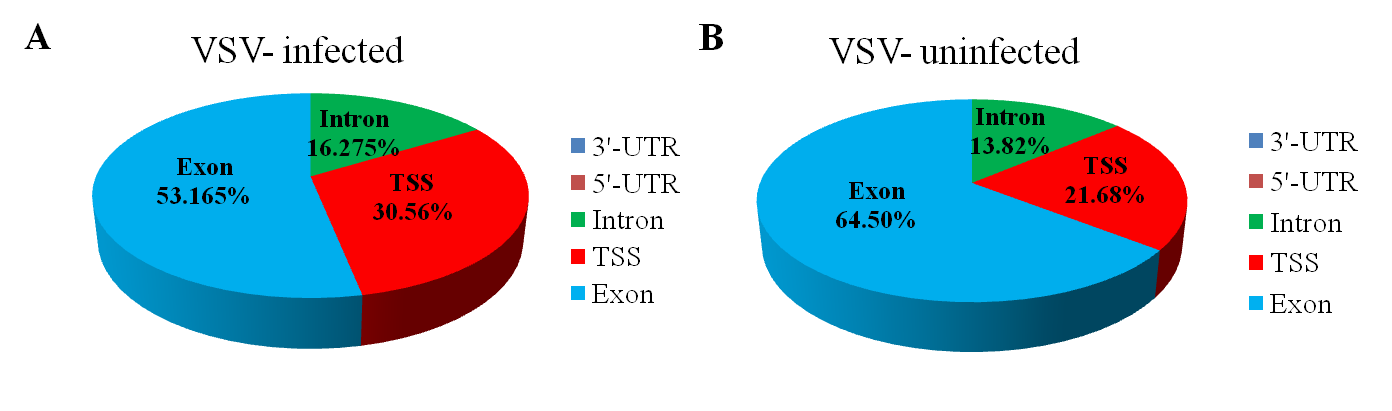

Supplement: S3 Fig — Frequency of total peaks under substantial iCLIP-seq date in MEF left uninfected or infected with VSV (MOI = 10). UTR, untranslated region; TSS, transcriptional start site. (TIF) [file ppat.1009530.s003.tif]

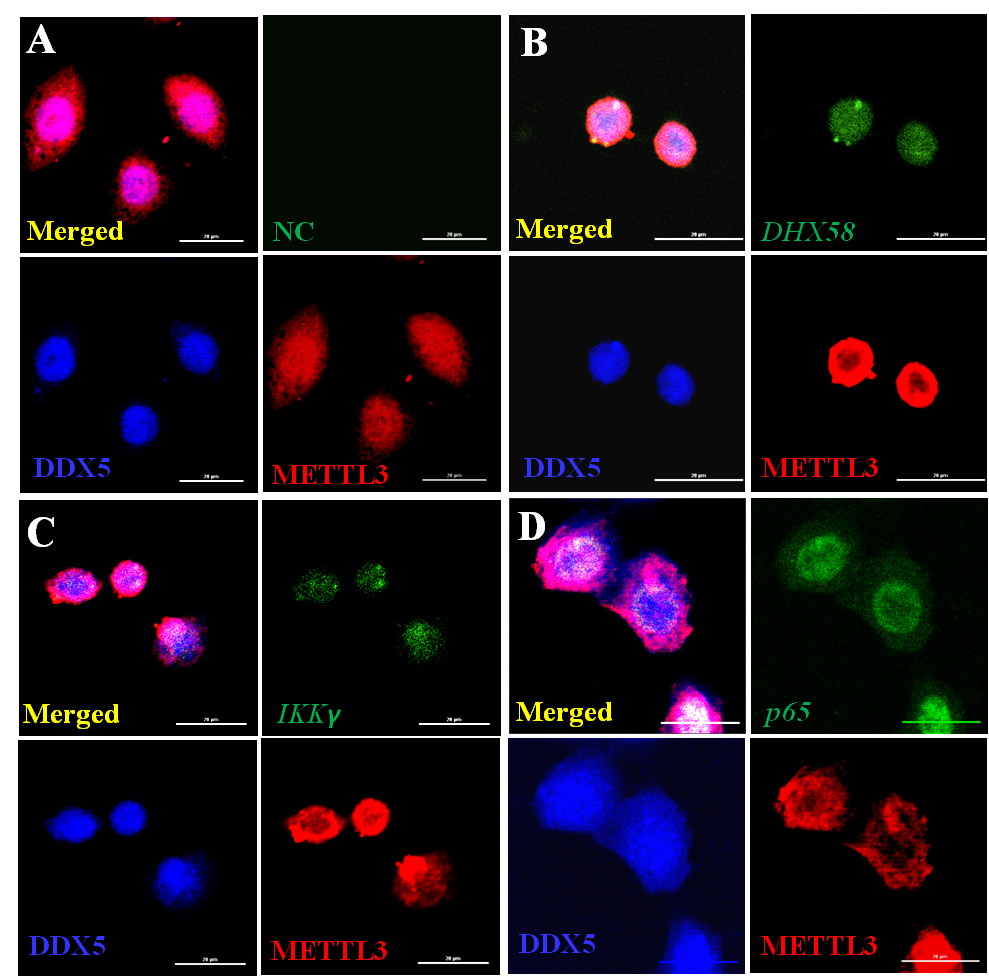

Supplement: S4 Fig — MEFs were infected with VSV (MoI = 10) for 6 h and fixed in 4% paraformaldehyde, the probes of trancripts were labeled with FAM using a FISH SA-Biotin system to suffer from IF combined with FISH assay. The transcripts were labeled with the probes, and the DDX5 protein and the METTL3 protein were labeled with mouse METTL3 monoclonal antibody and rabbit DDX5 monoclonal antibody following with goat anti-rabbit IgG (H+L) secondary antibody, DyLight 405 and goat anti-mouse IgG (H+L) highly cross-adsorbed secondary antibody, Alexa Fluor 647, then images were obtained with a CLSM. DDX5 was labeled with DyLight 405 fluorescent (Blue), METTL3 was labeled with Alexa Fluor 647 fluorescent (Red), and the transcripts were labeled with FAM-Probes (Green). A: RNA FISH negative control (NC); B: DHX58; C: IKKγ; D: p65. Scale bars, 20 μm. (TIF) [file ppat.1009530.s004.tif]

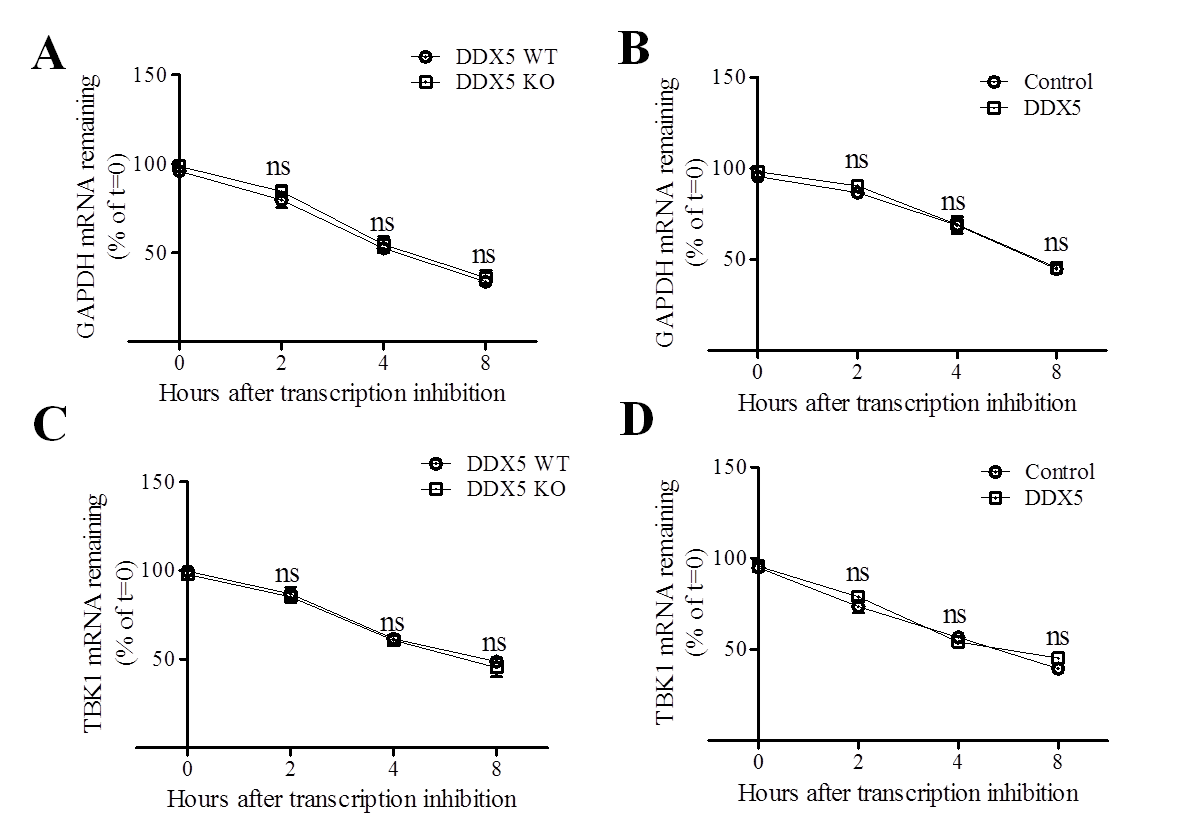

Supplement: S5 Fig — GAPDH mRNA degradation in VSV infected DDX5-knockout MEFs(A) or overexpressed MEFs (B) treated with actinomycin D at indicated times (n = 3). TBK1 mRNA degradation in VSV infected DDX5-knockout MEFs(C) or overexpressed MEFs (D) treated with actinomycin D at indicated times (n = 3). Residual RNAs were normalized to 0 h. (TIF) [file ppat.1009530.s005.tif]

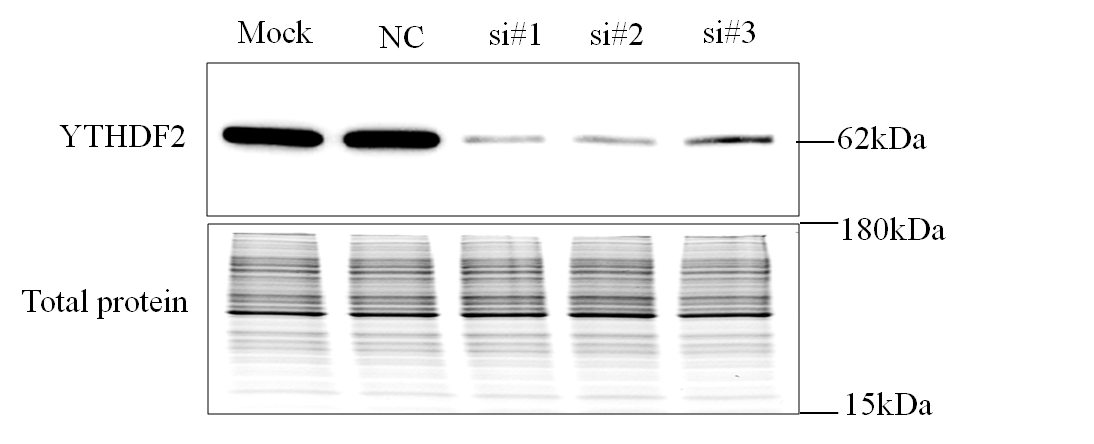

Supplement: S6 Fig — Knockdown effects of DDX5 were analyzed by immunoblot in MEFs transfected for 48 h with non-targeting control siRNA (NC) or YTHDF2-specific siRNA (siYTHDF2 (one of three siRNA constructs [si#1, si#1 and si#1]). (TIF) [file ppat.1009530.s006.tif]

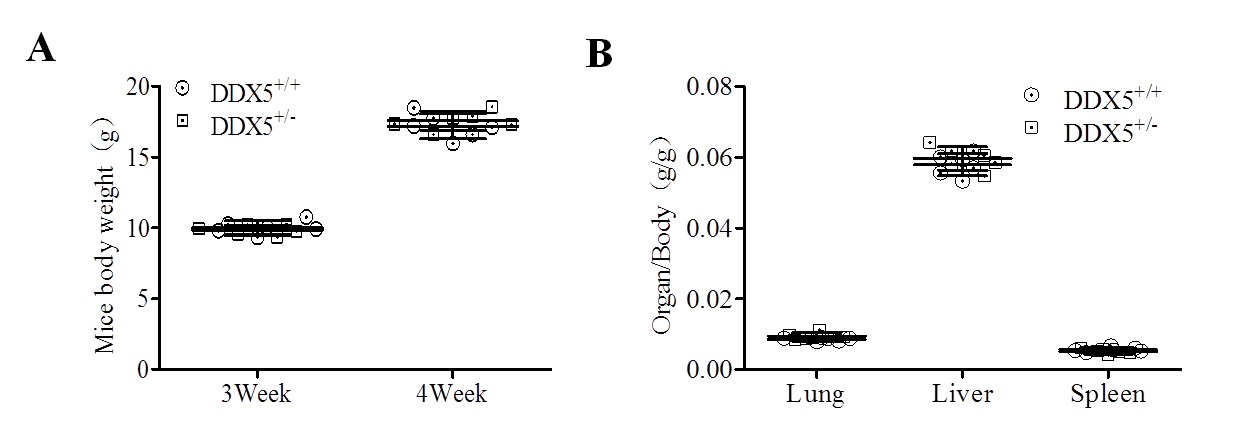

Supplement: S7 Fig — A: Quantification of DDX5+/+ or DDX5+/- mice at postnatal 3 and 4 weeks (n = 6). B: Organ-body rate was quantified between DDX5+/- mice and wild-type littermates (n = 6). (TIF) [file ppat.1009530.s007.tif]
