# Supplementary material for: The RNA helicase DDX5 promotes viral infection via regulating N6-methyladenosine levels on the DHX58 and NFκB transcripts to dampen antiviral innate immunity
Source: PLoS Pathog. 2021 Apr 28;17(4):e1009530. doi: 10.1371/journal.ppat.1009530 (PMC8081163; doi:10.1371/journal.ppat.1009530)
Supplement: S1 Table — (DOCX) [file ppat.1009530.s008.docx]

**S1 Table RNA oligonucleotides used for the depletion of cell genes in this study.**

| **Number** | **SiRNA sequence (5’to 3’)** |
| --- | --- |
| **1.Negative control(siNC)**  Sense  Anti sense  **2. siDDX5**  Sense  Anti sense  **3. siMETTL3**  Sense  Anti sense  **4. siYTHDF2**  #1 Sense  #1 Anti sense  #2 Sense  #2 Anti sense  #3 Sense  #3 Anti sense | UUCUCCGAACGUGUCACGUTT  ACGUGACACGUUCGGAGAATT  GCACAAUGGUAUGAACCAATT  UUGGUUCAUACCAUUGUGCTT  CCUCCAAGAUGAUGCACAUTT  AUGUGCAUCAUCUUGGAGGTT  GGUAGCACAGAAGUUGCAATT  UUGCAACUUCUGUGCUACCTT  GCAGUGGGUUCGGUCAUAATT  UUAUGACCGAACCCACUGCTT  GGGAUUGACUUCUCAGCAUTT  AUGCUGAGAAGUCAAUCCCTT |
