# Supplementary material for: The RNA helicase DDX5 promotes viral infection via regulating N6-methyladenosine levels on the DHX58 and NFκB transcripts to dampen antiviral innate immunity
Source: PLoS Pathog. 2021 Apr 28;17(4):e1009530. doi: 10.1371/journal.ppat.1009530 (PMC8081163; doi:10.1371/journal.ppat.1009530)
Supplement: S2 Table — (DOCX) [file ppat.1009530.s009.docx]

**S2 Table Primers used for gene cloning.**

| Genes Primer sequences (5’ to 3’) | |
| --- | --- |
| DDX5  (1-316AA) | Forward ATGTCCAGCTACAGCTCCGATAGGG |
|  | Reverse TTA GATCTGCAGGATGTTGTGGTTGGCG |
| DDX5  (1-496AA) | Forward ATGTCCAGCTACAGCTCCGATAGGG |
|  | Reverse TTA TCTATCCCTCCTATCATCCTTCATTC |
| DDX5  (1-532AA) | Forward ATGTCCAGCTACAGCTCCGATAGGG |
|  | Reverse ATTA GTTCTGGGTCTTGGCGCCGAAGTCCC |
| DDX5  (1-583AA) | Forward ATGTCCAGCTACAGCTCCGATAGGG |
|  | Reverse TTA TGTTGGCCACGTTGGAGCCGTACTGC |
| DDX5  (317-615AA) | Forward ATG GTGGACGTGTGCCACGATGTGGAGAAG |
|  | Reverse TTACTGGGAGTAGCCTGTAGGCATAG |
| DDX5  (497-615AA) | Forward ATG TACTCCGCCGGCAAGAGAGGCGGCTTC |
|  | Reverse TTACTGGGAGTAGCCTGTAGGCATAG |
| DDX5  (533-615AA) | Forward ATGGGCGTGTACTCCGCCGCCAACTACAC |
|  | Reverse TTACTGGGAGTAGCCTGTAGGCATAG |
| DDX5  (584-615AA) | Forward ATG ATGCACAACGGCATGAACCAGCAGGC |
|  | Reverse TTACTGGGAGTAGCCTGTAGGCATAG |
| METTL3  (1-550AA) | Forward ATGTCGGACACGTGGAGCTCTATCCAGGC |
|  | Reverse TTATTGGTTTCCAAGAGTAATCCAGTTGGGCTG |
| METTL3  (389-581AA) | Forward ATGTTTGCAGTTGTGATGGCTGACCCACCTTG |
|  | Reverse TTACTATAAATTCTTAGGTTTAGAGATGATGC |
